# Source localized infraslow neurofeedback training in people with chronic painful knee osteoarthritis: A randomized, double-blind, sham-controlled feasibility clinical trial

**DOI:** 10.3389/fnins.2022.899772

**Published:** 2022-07-28

**Authors:** Jerin Mathew, Divya Bharatkumar Adhia, Mark Llewellyn Smith, Dirk De Ridder, Ramakrishnan Mani

**Affiliations:** ^1^Centre for Health, Activity, and Rehabilitation Research, School of Physiotherapy, University of Otago, Dunedin, New Zealand; ^2^Department of Surgical Sciences, Dunedin School of Medicine, University of Otago, Dunedin, New Zealand; ^3^Neurofeedback Therapy Services, New York, NY, United States

**Keywords:** infraslow oscillation, knee osteoarthritis, neurofeedback, pain neuromodulation, safety, feasibility

## Abstract

Persistent pain is a key symptom in people living with knee osteoarthritis (KOA). Infra-slow Neurofeedback (ISF-NF) training is a recent development focusing on modulating cortical slow-wave activity to improve pain outcomes. A parallel, two-armed double-blinded, randomized sham-controlled, feasibility clinical trial aimed to determine the feasibility and safety of a novel electroencephalography-based infraslow fluctuation neurofeedback (EEG ISF-NF) training in people with KOA and determine the variability of clinical outcomes and EEG changes following NF training. Eligible participants attended nine 30-min ISF-NF training sessions involving three cortical regions linked to pain. Feasibility measures were monitored during the trial period. Pain and functional outcomes were measured at baseline, post-intervention, and follow-up after 2 weeks. Resting-state EEG was recorded at baseline and immediate post-intervention. Participants were middle-aged (61.7 ± 7.6 years), New Zealand European (90.5%), and mostly females (62%) with an average knee pain duration of 4 ± 3.4 years. The study achieved a retention rate of 91%, with 20/22 participants completing all the sessions. Participants rated high levels of acceptance and “moderate to high levels of perceived effectiveness of the training.” No serious adverse events were reported during the trial. Mean difference (95% CI) for clinical pain and function measures are as follows for pain severity [active: 0.89 ± 1.7 (−0.27 to 2.0); sham: 0.98 ± 1.1 (0.22–1.7)], pain interference [active: 0.75 ± 2.3 (−0.82 to 2.3); Sham: 0.89 ± 2.1 (−0.60 to 2.4)], pain unpleasantness [active: 2.6 ± 3.7 (0.17–5.1); sham: 2.8 ± 3 (0.62–5.0)] and physical function [active: 6.2 ± 13 (−2.6 to 15); sham: 1.6 ± 12 (−6.8 to 10)]. EEG sources demonstrated frequency-specific neuronal activity, functional connectivity, and ISF ratio changes following NF training. The findings of the study indicated that the ISF-NF training is a feasible, safe, and acceptable intervention for pain management in people with KOA, with high levels of perceived effectiveness. The study also reports the variability in clinical, brain activity, and connectivity changes following training.

## Introduction

Persistent pain is a significant presenting complaint in knee osteoarthritis (KOA), leading to activity limitation and participation restrictions (Vongsirinavarat et al., 2020). Available non-pharmacological and pharmacological interventions for reducing pain and improving function (Yusuf, 2016; Ferreira et al., 2019) only achieve modest improvements in pain outcomes and associated disability (Beswick et al., 2012; Kittelson et al., 2014; Gregori et al., 2018). Neuroimaging studies demonstrate altered cortical activities at the somatosensory cortex (SSC) (Gingold et al., 1991; Price, 2000; Craig, 2002; Bushnell et al., 2013), dorsal anterior cingulate cortex (dACC), (Rainville et al., 1997; Price, 2000; Vogt and Sikes, 2000; Rainville, 2002; Fallon et al., 2015; Vanneste et al., 2017), and the pregenual anterior cingulate cortex (pgACC) in persistent pain conditions including KOA (Price, 2000; Fields, 2004; Sofat et al., 2011; Bushnell et al., 2013; Kwon et al., 2014; Ossipov et al., 2014; Fingleton et al., 2015; Vanneste et al., 2017; Peters et al., 2019; Soni et al., 2019; Apkarian, 2020). A heuristic pathophysiological model suggests that pain is the consequence of an imbalance between the pain-evoking and descending pain inhibitory brain regions in patients with chronic pain treated by spinal cord stimulation (De Ridder and Vanneste, 2016, 2021; Vanneste and De Ridder, 2021).

Electroencephalography-based neurofeedback (EEG-NF) is a non-invasive neuromodulatory technique in which cortical electrical activity is measured and fed back in real-time to the individual to facilitate self-regulation of the cortical activity to influence specific behavior or clinical outcomes (Ros et al., 2014; Sitaram et al., 2017). EEG-NF protocols are designed either to upregulate or downregulate the cortical potentials of various regions of interest (ROI) linked to symptom or disease states (Rance et al., 2011; Gorini et al., 2015; Yang L. et al., 2015; Marzbani et al., 2016; Enriquez-Geppert et al., 2017; Shoji et al., 2017; Sitaram et al., 2017; Prinsloo et al., 2018; Reiner et al., 2018; Saj et al., 2018; Roy et al., 2020). The functional magnetic resonance imaging (fMRI) Blood Oxygenation Level Dependent (BOLD) signal fluctuates in the infraslow frequency (ISF) band (0.01–0.1 Hz) and correlates with EEG infraslow fluctuations (Pan et al., 2013; Hiltunen et al., 2014; Thompson et al., 2014; Keinänen et al., 2018; Gutierrez-Barragan et al., 2019). Increasing evidence implicates the role of a novel ISF in modulating dynamic brain connections (Ploner et al., 2017) and physiological and pathological brain functions including in chronic pain (Vanhatalo et al., 2004; Lőrincz et al., 2009; Schroeder and Lakatos, 2009; Hughes et al., 2011; Ko et al., 2011; Rodin et al., 2014). EEG-NF training targeting the ISF band (0.0–0.1 Hz) can produce clinical benefits (Leong et al., 2018; Balt et al., 2020). The ISF band correlates with higher frequency oscillations and is phase-locked with the faster frequency spectrum greater than 1 Hz extending to 20 Hz (Vanhatalo et al., 2005; Ploner et al., 2017). Preclinical and empirical research shows that neuropathic pain is associated with altered ISF within the dorsal horn, extending to the SSC (Gerke et al., 2003; Iwata et al., 2011). Additionally, evidence from neuroimaging studies demonstrates increased ISF activity within the pain-evoking brain regions, including dACC and SSC, and decreased ISF activity within the antinociceptive network, including pgACC (Majeed et al., 2011; Kucyi et al., 2013; Kucyi and Davis, 2015; Alshelh et al., 2016; Alshelh, 2018; Zhou et al., 2018; Zhang B. et al., 2019; Zhang Y. et al., 2019; Di Pietro et al., 2020). Also, greater infra-Slow oscillation power in the contralateral orbitofrontal cortex, insula, thalamus, secondary SSC, and the ipsilateral anterior insula was identified in individuals with CRPS compared with controls (Di Pietro et al., 2020). EEG-NF training of the ISF band can modulate the resting-state brain networks, and this has been demonstrated in both clinical and preclinical studies (Monto et al., 2008; Dong et al., 2012; Mairena et al., 2012; Smith et al., 2014). Currently, there is no evidence of the efficacy of ISF-NF training in people with pain. Moreover, there is no evidence on the safety, feasibility, and acceptability of the ISF-NF training as an intervention for persistent pain (Mathew et al., 2020). Therefore, the objectives of the study were: (1) to assess the feasibility, safety, and acceptability of ISF-NF training in individuals with KOA; (2) to descriptively report the variability of change in the clinical and experimental pain outcomes in people with KOA following NF training; (3) to descriptively explore the changes in EEG current source density (CSD) at the targeted cortical areas (SSC, dACC, and pgACC), functional connectivity between the three cortical areas (SSC, dACC, and pgACC), and the CSD ratios between the ROIs (SSC, dACC, and pgACC) following ISF-NF training.

## Materials and methods

### Design, ethics, and cultural consultation

A two-arm, parallel-group, double-blinded, randomized sham-controlled feasibility trial was conducted in Dunedin, New Zealand. The trial is reported according to the Consolidated Standards of Reporting Trials (CONSORT) extension for randomized pilot and feasibility trials (Eldridge et al., 2016). The description of the study intervention was structured following the Template for Intervention Description and Replication (TIDieR) guide (Hoffmann et al., 2014). The trial was registered with the Australian New Zealand Clinical Trials Registry (ACTRN12620000273987), and the study protocol was published in a peer-reviewed journal (Mathew et al., 2020). This study was approved by the Health and Disability Ethics Committee (HDEC), New Zealand (19CEN182), and the cultural consultation was obtained from the Ngāi Tahu Research Consultation Committee (5733_21392).

### Sampling and recruitment strategy

Study participants were recruited from the Dunedin community using convenience sampling. Periodic advertising was carried out through Newspapers, Facebook (Sponsored), an e-mail invitation to the University of Otago (UO), displaying study flyers at the Public Hospital, School of Physiotherapy clinic, and other UO departments. Interested volunteers contacted the first author via telephone or email and underwent screening using the Qualtrics online survey platform (Qualtrics, Provo, UT, United States, 2013) (Cantor and Thorpe, 2018). Eligible participants were further contacted via phone, and appointments were fixed for the confirmatory baseline assessment session. Eligible participants provided written consent and completed a baseline assessment. Calendar invitations were sent to all the participants confirming their appointments for their training sessions and post-intervention assessment session. Automated reminder emails or text messages were sent prior to assessment and training session days.

#### Inclusion criteria

Adults aged 44–75 years, with a clinical diagnosis of KOA with pain severity of at least ≥ four on an 11-point numerical rating scale for a minimum duration of 3 months were eligible to participate in the study (Fingleton et al., 2015; Bartley et al., 2016).

#### Exclusion criteria

Participants were excluded if they had one of the following situations or conditions: underwent surgery or other invasive procedures in the last 6 months and any surgical procedures scheduled within 8 weeks after screening; undertaken any steroid injections to the knee joint in the past 3 months or on oral steroids in the previous month; current intake of centrally acting medications or intention of taking new medications in the next 8 weeks; neurological conditions or diseases; soft tissue injuries of the knee in the last 3 months; cognitive impairments; difficulty or inability to read or understand English or provide informed consent; and pregnancy or 6 months post-labor. A paper-based Mini-Mental State Examination (MMSE) was carried out for screening volunteers with cognitive impairments. The maximum MMSE is scored out of 30 points, and volunteers scoring a total score of 24 or below were excluded from the study.

### Randomization and allocation concealment

Participants were randomized into either active ISF-NF or sham ISF-NF groups (in a ratio of 1:1) using an open-access block randomization program by the department research administrator not involved in the assessments, allocation, or interventions. The allocations were concealed until after the initial assessment was completed. The NF provider (first author) opened the sealed envelope before the first NF training session.

### Blinding

Participants and the outcome assessor were blinded to the group allocation. The group was disclosed to the participant after completing the follow-up questionnaire after 2 weeks after the post-intervention assessment session. Every week, all the participants were asked at the end of the third training session, “Which training condition do you think you received?” to determine the blinding integrity (Leong et al., 2018).

### Primary study outcomes

#### Feasibility outcomes

The *recruitment rate* was determined by the number of participants attending the screening assessment. The *randomization rate* was assessed by the ratio of the number of participants randomized into the trial from amongst those eligible. The *retention rate* was determined by the number of sessions attended by the participant as per the initial appointment schedule. The *drop-out rate* was measured as the number of participants who dropped out from each group and expressed as a percentage of the total number of participants enrolled in the study.

##### Acceptability

Acceptability is a “multi-faceted construct that reflects the extent to which people receiving a healthcare intervention consider it appropriate, based on anticipated or experienced cognitive and emotional responses to the intervention” (Sekhon et al., 2017). All the participants reported the training acceptability on a Likert scale with “0” corresponding “not at all acceptable” and “7” corresponding to “very acceptable” on their post-intervention assessment.

##### Perceived level of effectiveness

The subjective likelihood that the ISF-NF training will have a persuasive impact on the participant (Suka et al., 2018). Participants reported their perceived levels of effectiveness on a Likert scale with “0-not at all effective and 7-very effective” on their post-intervention assessment.

#### Adverse events

An adverse effect is described as any harmful sign or symptom resulting from the treatment, which could be related to the ISF-NF training. All the participants were instructed to complete a Discontinuation-Emergent Sign and Symptom (DESS) inventory to record worsening or improving side effects compared to the status before every training session. DESS has been used in previous NF studies to record adverse events associated with NF training (Rogel et al., 2015). DESS is a checklist of 43 symptoms, consisting of emotional, behavioral, cognitive, and physical conditions that can be considered possible side effects from NF training. At each training session, participants were asked the following question: “Since the last visit, have you experienced any changes in the following symptoms? (Please check only one response for each symptom).” The scale was originally developed and used to capture symptoms associated with discontinuation or interruption of Selective Serotonin Reuptake Inhibitors (SSRI) treatment (Bhat and Kennedy, 2017). Participants rated each symptom as a “new symptom,” “old symptom but worse,” “old symptom but improved,” “old symptom but unchanged,” and “symptom not present.” We have reported any adverse effect if the participant has reported any symptom as a “new symptom” at least once during the trial, which they think is related to the NF training.

### Secondary outcome measures

Pain, function, and psycho-social constructs were collected using validated questionnaires. The multi-dimensional constructs were chosen based on the biopsychosocial model of pain literature. Based on the recommendations by Initiative on Methods, Measurement, and Pain Assessment in Clinical Trials (IMMPACT) consensus II, Pain (both numerical and categorical tools), physical and emotional functioning measurement tools were included (Edwards et al., 2016). A summary of the outcome measures is given in Supplementary Table 1. Details including psychometric properties and the method of implementation of each tool are explained in the protocol (Mathew et al., 2020).

#### Pain sensitivity

Quantitative Sensory Testing (QST) procedures including pressure pain threshold (PPT), mechanical temporal summation (MTS), conditioned pain modulation (CPM), vibration perception threshold, cold hyperalgesia, tactile acuity, and body schema integrity (Rolke et al., 2006; Georgopoulos et al., 2019) are detailed in the published protocol (Mathew et al., 2020) and are also summarized in (Supplemental Digital Content).

#### EEG and source localization

Resting-state EEG was obtained in a quiet room while the participant was sitting upright in a comfortable chair by an independent researcher. EEG was collected using the Mitsar EEG system sampled at 500 Hz with WinEEG software at baseline (T0) and immediately post-intervention (T1) (Gorecka and Makiewicz, 2019). The EEG was sampled with 21 electrodes placed in the standard 10–20 International placement, and impedances were checked to remain below 5 kΩ (Khazi et al., 2012). Data was collected for ∼10 min with the participant’s eyes closed. The participants’ alertness was observed by monitoring both the slowing of the alpha rhythm and the appearance of spindles in the EEG stream to prevent possible enhancement of the theta power due to drowsiness during recording (Britton et al., 2016).

#### Follow-up assessment

Two weeks following the final training session, all participants were contacted by a phone call or email to complete the online survey on pain (pain intensity, pain bothersomeness, pain unpleasantness) and adverse events using the DESS tool.

### Interventions

Eligible participants attended nine sessions (30 min each; three sessions/week) of NF training at the School of Physiotherapy, UO. The intervention was provided by a researcher (JM) who was not involved in the outcome assessment and randomization process. Two NF experts trained the researcher who provided the intervention in the trial. Before the trial, the researcher conducted NF training sessions on 10 healthy people to get familiarized with standardized training protocols (Mathew et al., 2020). The experimental methodology and reporting of NF followed the Consensus on the Reporting and Experimental Design of clinical and cognitive-behavioral NeuroFeedback studies checklist (CRED-nf checklist) (Ros et al., 2020). Since this was a feasibility trial, most of the items on the CRED-nf list were not applicable.

During each session, participants were asked to sit on a chair with their back supported and stay relaxed for 10 min, allowing the trainer to prepare the participant for the NF training. Both active ISF-NF and sham ISF-NF were administered using a 21-channel DC-coupled amplifier produced by BrainMaster Technologies, Inc. The amplifier was connected to a high-end laptop (G752VS, Intel^®^ Core™ i7-6700HQ CPU @ 2.60GHZ) produced by the ASUSTek computer INC. The Comby EEG lead cap with sensors (Ag/AgCl) with appropriate size was fixed to the individual’s head, with reference electrodes placed at the mastoids. A sparse amount of EEG gel was applied to each electrode using a syringe with caution to prevent bridging between adjacent electrodes. The tip of the syringe was used to move the hair beneath each electrode, and a mild abrasion of the scalp was performed. This was to ensure proper electrode placement on the scalp and to acquire quality EEG recording.

The impedance of the active electrodes was monitored and kept below 5 kΩ (Leong et al., 2018). The optimal impedance was acquired by manual adjustment of the electrode initially, and an additional amount of gel was applied, if necessary, but always sparsely. The trainer ensured no more than 5 ml of the gel had been utilized for each training preparation. Any amount of gel escaping from the electrode was removed using a cotton swab before the commencement of the training session. The NF training was performed in a large, closed room with appropriate ventilation. All efforts were made to maintain the room temperature with a central temperature control system. A silent cooler was used when necessary. This was to control (not eliminate) the influence of sweat with EEG recording. The room lighting and influence of external sound were controlled during each training session. Clear instructions were given to the participant before starting the NF training. Participants were emphasized to minimize eyeball movement, head and neck movements, swallowing, and clenching of teeth to minimize motion artifact in the EEG. Each NF session consisted of 30 min of training. The following instruction was given to all the participants before every NF session; *“please close your eyes, keep your eyes and tongue nice and still. Listen and concentrate on the sound being played for the coming 30 min. Please let me know if you experience any problem or discomfort; I am right behind you controlling the training system.”*

#### Active infraslow neurofeedback balance training protocol

**FIGURE 1:**
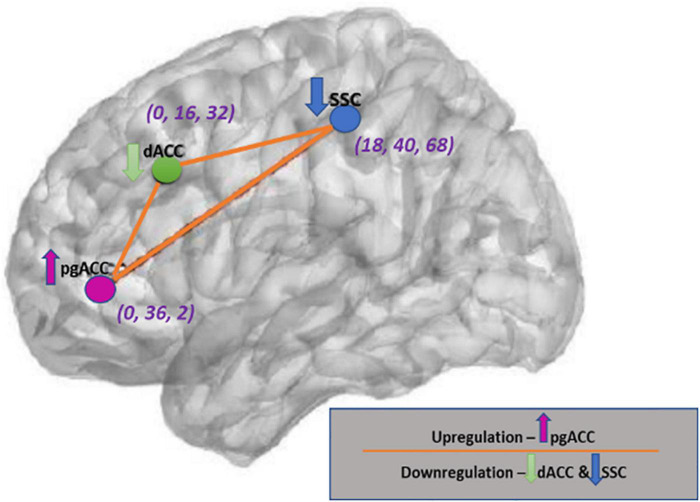
The Regions of interests and neurofeedback training directions with respective coordinates (XYZ). This figure was created using brain images produced from eLORETA software and with the help of Microsoft PowerPoint application.

A novel ISF-NF balance training protocol was developed to enhance a balance between the three cortical areas (SSC, dACC, and pgACC) (De Ridder and Vanneste, 2020). The idea of ISF-NF balance training protocol was based on the previous studies that observed an imbalance between the dACC and SSC and pgACC cortical areas (De Ridder and Vanneste, 2020; Vanneste and De Ridder, 2021). The ISF-NF training protocol (Figure 1) involved simultaneously downregulating the electrical activities of SSC (sensory-discriminative function) and dACC (motivational/affective function), and upregulating the pgACC (descending nociceptive inhibitory function) (Rainville et al., 1997; Vogt and Sikes, 2000; Rainville, 2002; Kulkarni et al., 2007; Baliki et al., 2008, 2014; Kong et al., 2010; Tagliazucchi et al., 2010; Howard et al., 2012; Bushnell et al., 2013; Kucyi et al., 2013; Kwon et al., 2014; Ossipov et al., 2014; Fallon et al., 2015; Vanneste et al., 2017; Cottam et al., 2018; Furman et al., 2018; Yam et al., 2018; De Ridder and Vanneste, 2020; Spisak et al., 2020). The SSC was made up of Brodmann areas 1, 2, 3, and 5, as defined by the Montreal Neurological Institute (MNI) coordinate database (Fuchs et al., 2002; Lancaster et al., 2007). The dACC and pgACC were designer ROIs and are defined with the help of the NeuroSynth meta-analytic database.^1^

Each participant received auditory feedbacks delivered by BrainMaster Technologies Software for an approximate of 60–80% in real-time when the participant’s brain activity met the desired infraslow (0.0–0.1 Hz) threshold at the targeted ROIs.

The software calculated the ratio in real-time in the ISF band, and the feedback was given when the ratio was > 1 based on satisfying the following equation:


$$\frac{2*p\,g\,A\,C\,C}{S\,S\,C+d\,A\,C\,C}=1$$

As the dACC and SSC combine two current densities and the pgACC only one, the current density of the pgACC was doubled (Vanneste and De Ridder, 2021). Efforts were made to keep the reward threshold between 60 and 80%. In other words, 60–80% of the time, a sound will be played (reward) when the participant’s brain activity meets the infraslow magnitude (threshold). This was chosen based on the insights from our previous study and the authors’ clinical experience (Leong et al., 2018). Reaching a predetermined threshold brain activity (activities) is a response in relation to the received feedback and participant’s engagement with the training (Enriquez-Geppert et al., 2017; Sitaram et al., 2017). The software delivered the auditory feedback within 30 ms when the activity threshold is met (upregulation of pgACC and downregulation of SSC and dACC). However, further improvement in the response would be dependent on how the participant responds to the reinforcement (Sitaram et al., 2017).

#### Sham infraslow neurofeedback protocol

Conditions for the sham ISF-NF group was the same as the active ISF-NF group, except the participants received sound feedback according to someone else’s pre-recorded session. To ensure this, we have trained healthy participants with an active ISF-NF program for nine sessions. We captured the feedback sound using Audacity software, a free and open-source digital audio editor and recording application (Maheshkumar et al., 2016). Participants in the sham ISF-NF were prepared as same as the ISF-NF group, and they received these pre-recorded feedback sounds. The Audacity software uses the computer’s sound card as an audio to digital (A/D) converter and eliminates the additional requirement of an external microprocessor (Maheshkumar et al., 2016). The pre-recorded signals were selected randomly by the chit method from a set of nine files.

## Data processing

### EEG data processing

Using WinEEG advanced software, raw EEG data were sampled at 500 Hz for 19 channels, filtered from DC to 50 HZ, plotted, and exported in American Standard Code for Information Interchange (ASCII) format (Golomolzina et al., 2014).

The exported files were then batch imported into the EEGLAB (MATLAB R2020a). Each file was resampled to 128 Hz, bandpass filtered from 0.01 to 0.1 Hz and the first 4 s of data were truncated. Subsequently, Average Fourier cross-spectral matrices (Lin and Cai, 2002) were computed for the infraslow band (0.01–0.1 Hz). The transformation matrix was exported for import into the Independent Component Neurofeedback (ICoN) software (Delorme and Makeig, 2004). The ROIs used in the present study were SSC left (S1Lt) *[MNI coordinates (MNIxyz) = 18, −40, 68]*, SSC right (S1Rt) *(MNIxyz = −18, −40, 68)*, pgACC *(MNIxyz = 0, 36, 2)*, and dACC *(MNIxyz = 0, 16, 32)* (Figure 1).

Each EEG file was carefully inspected in ICoN for eye blinks, muscle artifacts, perspiration, and body movements, and the artifacts were manually rejected from the file. ICoN performs fast blind source separation (BSS) on multiple EEG time-series using second-order statistics (SOS). The ICoN allows source estimation, data filtering (e.g., artifact rejection), and source localization of separated sources through the LORETA-Key software (Pascual-Marqui, 2002; Congedo et al., 2008). The software is widely used in EEG studies for BSS and artifact rejection (Bersagliere et al., 2018; Leong et al., 2018; Vanneste and De Ridder, 2021). The raw EEG data were randomized by the research administrator and the pre-processing and cleaning of the EEG data was blinded. The blinding was concealed until the EEG cleaning was completed.

### EEG based outcome measures

#### Region of interest analysis

Exact low-resolution brain electromagnetic tomography (eLORETA) software (Jatoi et al., 2014; Aoki et al., 2015) was used to perform a voxel-by-voxel analysis (comprising 6,239 voxels) for the ISF frequency band of the CSD distribution. The log-transformed current density was averaged across all voxels belonging to the S1Lt, S1Rt, dACC, and pgACC for the ISF frequency band to identify differences in brain electrical activity between the groups. Means, standard deviations, and mean differences for the CSD at the targeted ROIs in the ISF band were calculated.

We also computed the ISF band ratios between the ROIs as below and descriptively compared between the groups.


$$\frac{2*p\,g\,A\,C\,C}{S\,S\,C+d\,A\,C\,C}$$

#### Lagged phase coherence or functional connectivity analysis

Similar eLORETA techniques were applied to compute the FC between the ROIs (Pascual-Marqui, 2002). FC is a statistical measure of coherence, i.e., co-varying activity or phase synchronization between two areas in the brain (Vanneste et al., 2017; De Ridder and Vanneste, 2020). The lagged phase synchronization between S1Lt, S1Rt, pgACC, and dACC reflects the communication between those areas (Lancaster et al., 2007; Mathew et al., 2020). Measures of linear dependence (coherence) between the multivariate time series are defined. Functional connectivity contrast maps were calculated through multiple ROI-by-ROI comparisons in eLORETA. The significance threshold was based on a permutation test with 5,000 permutations. The log-transformed electric current density of the FC strength was derived for each connection between the ROI and presented descriptively. The source localization FC analysis is widely used in neuromodulation and neurophysiological studies (Pascual-Marqui, 2007, 2017; Duke Han et al., 2013; Yang X. et al., 2015; Fallon et al., 2016; Pascual-Marqui et al., 2018; Aedo-Jury et al., 2020; Guo et al., 2020; Modares-Haghighi et al., 2021).

All the graphs and calculations were performed using GraphPad Prism software version 9.1.0 for Windows (GraphPad Software, San Diego, CA, United States).

### Descriptive analysis

As this was a feasibility trial, a formal sample size estimation was not conducted, and feasibility outcomes were reported based on the feasibility trial recommendations (Tickle-Degnen, 2013; Orsmond and Cohn, 2015). Hypothesis testing to compare study groups was not performed; instead, data were reported descriptively in aggregate and group allocation. Means and standard deviations are reported for continuous normal distribution data. Medians and percentile range are reported for non-normal data. Counts and percentages are reported for categorical data. Both 95 and 75% confidence intervals (CI) for the mean differences were derived and reported for the pain and function measures. Based on the intention-to-treat analysis principle, a “last observation carried forward” methodology was used to impute the missing data for one participant for the follow-up assessment session (Moher et al., 2012; Twisk et al., 2020).

## Results

Twenty-two participants with KOA underwent baseline assessment and were randomly assigned to receive either active ISF-NF (*n* = 11) or sham ISF-NF (*n* = 11). Participants were middle-aged (61.7 ± 7.6 years), New Zealand European (90.5%), and mostly females (62%) with an average knee pain duration of 4.0 ± 3.4 years. The demographic and clinical characteristics of the participants at baseline are summarized in Table 1. Baseline measures of physical activity, quality of life, health status, psycho-social, and sleep measures are presented as Supplementary Table 2.

**TABLE 1:** Demographic and clinical characteristics of the participants.

Characteristics	Active group (*n* = 11)	Sham group (*n* = 10)
Age, years, M ± SD	62.3 ± 8.5	61.0 ± 6.7
Sex, n (%) Female	7 (64%)	6 (60%)
**Race, n (%)**		
New Zealand European	10 (91%)	9 (90%)
Australian	1 (9%)	0
Tongan	0	1 (10%)
Body Mass Index, kg/m^2^, M ± SD	32.4 ± 9.5	30.4 ± 8.8
**Employment, n (%)**		
Employed part-time	3 (27%)	3 (30%)
Employed full-time	2 (18%)	2 (20%)
Student	1 (9%)	0
Retired	4 (36%)	4 (40%)
Unemployed	1 (9%)	1 (10%)
**Education, n (%)**		
No formal qualifications	1 (9%)	0
Year 12 or equivalent (school certificate)	1 (9%)	0
Year 10 or equivalent (school certificate)	0	1 (10%)
Trade/apprenticeship	2 (18%)	2 (20%)
Certificate/diploma	3 (27%)	3 (30%)
University degree/higher university degree	4 (37%)	4 (40%)
Dominance, n (%) Right	11 (100%)	10 (100%)
Average pain in the last 3 months (on NPRS)	6.1 ± 1.5	5.9 ± 1.2
**Affected knee, n (%)**		
Right	8 (72.7%)	7 (70%)
Left	3 (27.3%)	3 (30%)
Knee pain duration–years (mean, SD)	5.3 ± 4	2.6 ± 2.3
**Current medication/treatment status**		
Analgesics	4 (36.4%)	6 (60%)
Physiotherapy	1 (9.1%)	1 (10%)
Analgesics and physiotherapy	2 (18.2%)	0
No-treatment	4 (36.4%)	3 (30%)

### Missing data

One person dropped out from the study during the training session and his data is not demonstrated in the results. Sport/recreational item was missing from the baseline and post-intervention assessment from one participant.

### Feasibility outcomes

#### Recruitment rate

**FIGURE 2:**
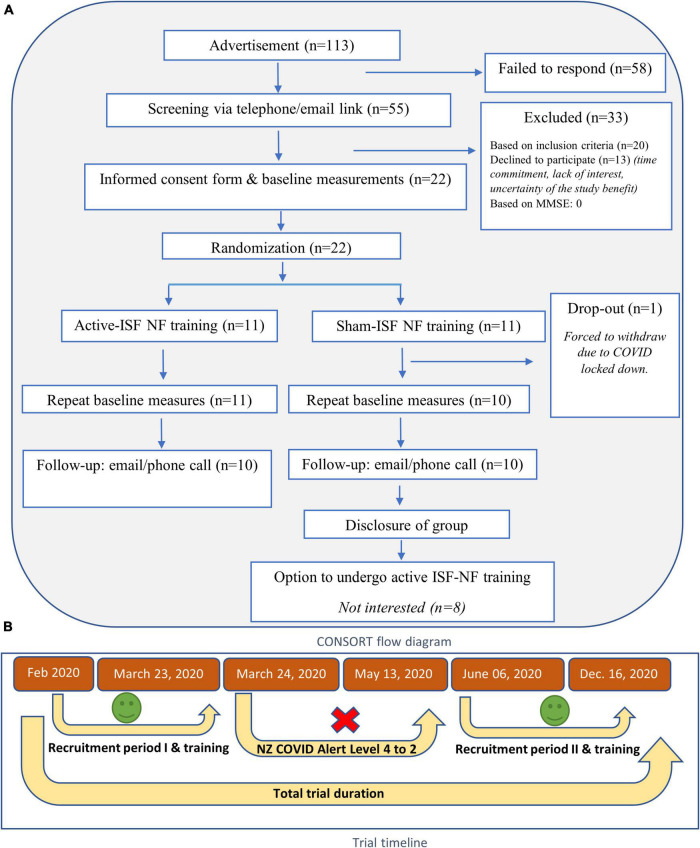
**(A)** CONSORT flow diagram **(B)** trial timeline.

The recruitment period was 5 months. A total number of 113 people indicated their interest to take part in the study. Of 113, 58 people could not be reached either via email or phone. Fifty-five participants completed online screening, out of which 20 did not meet the inclusion criteria, and 13 declined to participate in the study. Twenty-two participants attended baseline assessment sessions and were randomized into either active or sham groups. A detailed CONSORT flow diagram and trial timeline are given in Figures 2A,B. We were able to recruit an average of 4 participants per month for 5 months of the recruitment period. The details of recruitment and data collection are summarized in Table 2.

**TABLE 2:** Recruitment and data collection timeline.

	Feb. 2020			Mar	Apr	May	Jun		Aug		Oct	Nov	Dec
	NP	FB	UOM	NP			FB	UOM	NP	FB	NP		
Interests	14	3	5	17	COVID lock-down related interruption		10	12	14	16	22	NF training sessions continued.	
Eligible	4	0	2	6			0	1	6	0	8		
Baseline assessment	4	0	1	4			0	2	5	0	6		
Randomized	4	0	1	4			0	2	5	0	6		

NP, News paper; FB, Facebook; UOM, University of Otago e-mail circulation.

Table 2 summarizes the number of participants recruited through various advertisement strategies employed in this trial. The overall data collection period was from February to December 2020.

#### Randomization rate

None of the participants indicated any objection or raised concerns regarding the randomization process. All the participants who underwent baseline assessment sessions (*n* = 22) were randomized to receive either active ISF-NF training (*n* = 11) or sham ISF-NF training (*n* = 11).

#### Retention rate

The study achieved a 95.4% retention rate at the end of the trial, with 21/22 participants completing all the nine NF training sessions (S1–S9). However, 85.7% rescheduled their training sessions at least once during the NF training period due to various personal reasons.

#### Drop-out rate

Due to the COVID-19 lockdown in New Zealand (Susan Strongman et al., 2021), one participant in the sham group was forced to withdraw from the study after the seventh training session. Also, one participant from the active group failed to respond to the follow-up email and/or phone call.

### Acceptability and perceived levels of effectiveness

Both active and sham group participants reported ISF-NF training as an acceptable intervention with high levels of perceived levels of effectiveness (Table 3).

**TABLE 3:** Motivation and engagement levels and success of participant blinding.

	Active group (*n* = 11)	Sham group (*n* = 10)
**BCI-QCM-Motivation**		
**Baseline *M (SD)***		
Interest	4 (0.8)	3.9 (0.7)
Mastery of confidence	4.1 (0.5)	3.9 (0.9)
Incompetence fear	4.1 (0.8)	3.7 (0.7)
Challenge	2.8 (0.7)	2.3 (0.6)
**Before every NF session, *M (SD)***		
VAS-motivation	8.4 (1.3)	7.9 (1.4)
Mood-BMIS	8.1 (1.4)	8 (1.3)
**Level of engagement**		
**Post every NF session, *M (SD)***		
Level of engagement	8.6 (1.1)	8.3 (1.4)
**Post-training (9 sessions), *M (SD)***		
Treatment acceptability	6.3 (0.9)	6.5 (0.5)
Level of perceived effectiveness	4.8 (2.1)	5.7 (1.3)
**Blinding success, n (%)**		
End of week 1 (following S3), correct prediction	10 (90 %)	4 (40%)
End of week 2 (following S6), correct prediction	7 (64 %)	4 (40%)
End of week 3 (following S9), correct prediction	8 (73 %)	3 (30%)

BCI-QCM, Brain-computer interference-Questionnaire of current motivation; NF, Neurofeedback; VAS, Visual analog scale; BMIS, Brief mood introspection scale; S3, S6, S9, third/sixth/ninth NF session.

### Motivation, mood, and level of engagement

**FIGURE 3:**
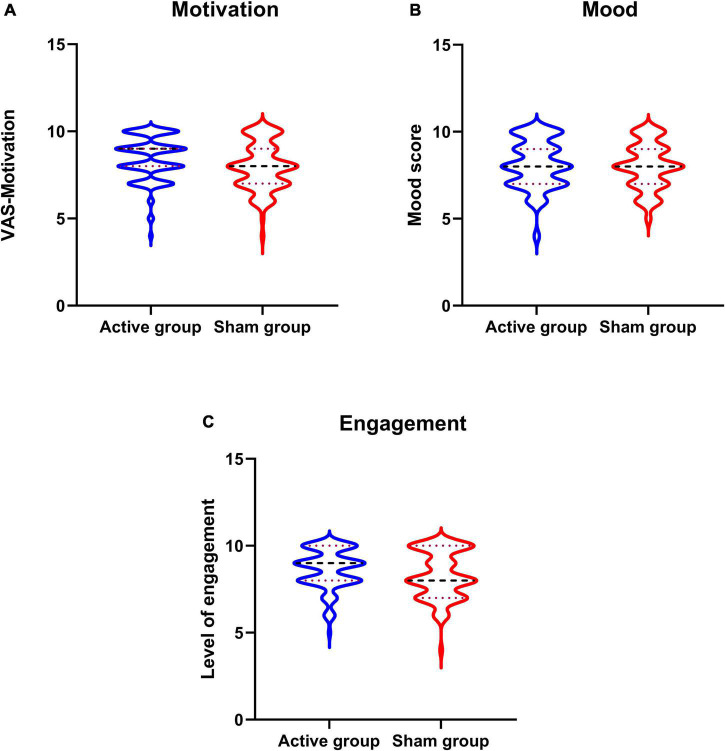
Overall motivation, mood, and engagement with neurofeedback training sessions. **(A–C)** Violine plot of participants motivation and mood recorded before every neurofeedback session and level engagement scores recorded after the sessions.

Participants in both the groups had reported higher levels of motivation, mood, and engagement with the training sessions (Table 3 and Figures 3A–C).

### Integrity of blinding

Eight participants from the active group correctly identified their allocation group at the end of the training sessions. The results are presented in Table 2. Out of eight, six reported improvements in pain and function as their reason for guessing the group allocation. Two of them were being hopeful and believed that they were in the active group. Only three participants from the sham group correctly identified their allocation group reported due to no improvement in their pain. Participants who failed to identify their allocation group correctly reported various reasons, including being hopeful, positive feeling, and pain improvement.

### Negative and positive events recorded using discontinuation-emergent sign and symptom scale

**FIGURE 4:**
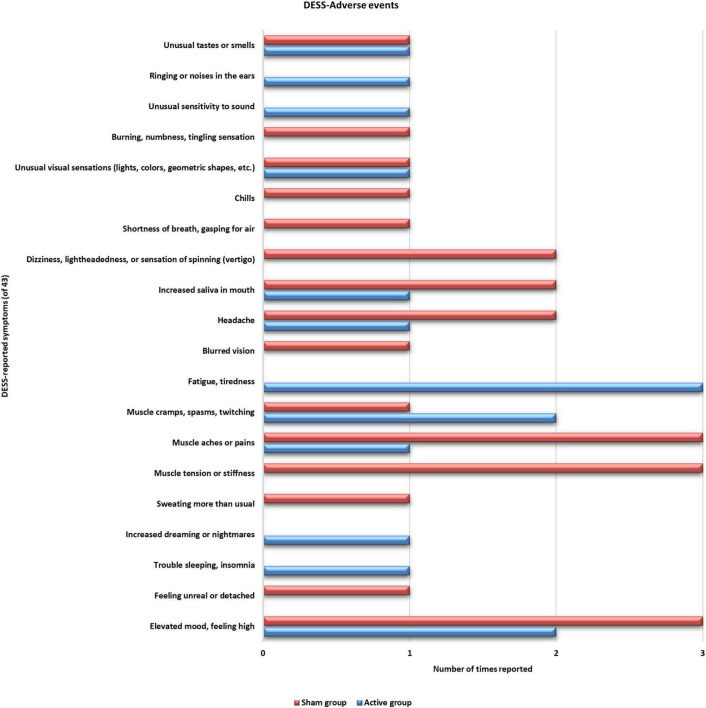
Distribution of the reported adverse effects by both groups on the Discontinuation-Emergent Sign and Symptom inventory.

No serious adverse effects were reported during the trial period. Also, none of the reported symptoms was worsened during the training period (Figure 4). Two sham group participants reported improvement (old symptom but improved) with their “nervousness or anxiety” symptoms after the 4th training session. Three participants from both groups reported improved sleeping after the 3rd and 4th training sessions. One sham group participant reported having improvement with “agitation” symptoms after the 3rd training session. One participant from active ISF-NF and two participants from sham ISF-NF reported improved “fatigue and tiredness” after the 3rd training session. The participants in both groups reported no other symptoms.

### Changes in the clinical outcome measures

**FIGURE 5:**
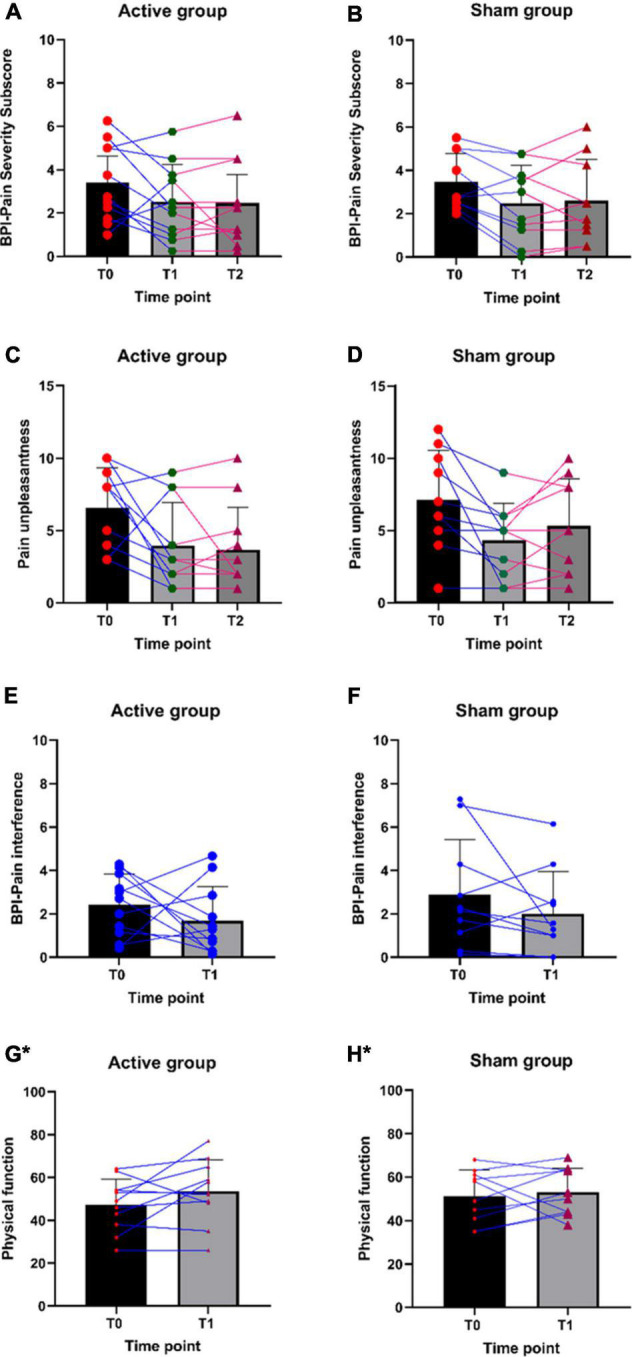
**(A–H)** Individual participant data for pain severity, pain unpleasantness, pain interference, and physical function scores. *Higher the score, the higher the physical function.

Participants in both groups have shown changes in pain severity, pain interference, and unpleasantness (Table 4). Individual participant data for all outcome measures are plotted in Figures 5A–H. Descriptive trend analyses show that the active group (vs. Sham group) showed greater improvement in pain unpleasantness, pain interference, physical function, and physical activity measures, and positive changes in the QST measures (Tables 4–6). These observations were made based on the descriptive data and graphical trend. No statistical analysis was conducted, as this was a feasibility trial and not powered to perform any statistical analysis.

**TABLE 4:** **Results of pain and function outcome measures**.

Domains and variables	Active group (*n* = 11)						Sham group (*n* = 10)					
	Baseline (T0)	Post-intervention (T1)	Follow-up (T2)	T0-T1	T0-T2	T1-T2	Baseline (T0)	Post-intervention (T1)	Follow-up (T2)	T0-T1	T0-T2	T1-T2
**Pain severity and interference -BPI, *M (SD)*, 95% CI and 75% CI**												
Pain severity sub-score (24 h items)	3.4 (1.8) [2.2,4.6] [2.7, 4.0]	2.5 (1.7) [1.3, 3.7] [1.9, 3.1]	2.4 (2.1) [1.1, 3.8] [1.7, 3.2]	0.89 (1.7) [−0.27, 2.0] [0.25, 1.5]	0.95 (1.5) [−0.064, 2.0] [0.40, 1.5]	0.07 (1.1) [−0.69, 0.83] [−0.35, 0.49]	3.4 (1.3) [2.5, 4.4] [2.9, 3.9]	2.5 (1.7) [1.2, 3.7] [1.8, 3.1]	2.6 (1.9) [1.2, 3.9] [1.8, 3.3]	0.98 (1.1) [0.22, 1.7] [0.56, 1.4]	0.85 (0.86) [0.24, 1.5] [0.52, 1.2]	−0.13 (0.98) [−0.83, 0.58] [−0.51, 0.26]
Pain interference sub-score	2.4 (1.4) [1.5, 3.4] [1.9, 2.9]	1.7 (1.6) [0.63, 2.7] [1.1, 2.3]	Not applicable (NA)	0.75 (2.3) [−0.82, 2.3] [−0.11, 1.6]	NA	NA	2.9 (2.5) [1.1, 4.7] [1.9, 3.9]	2.0 (1.9) [0.65, 3.4] [1.3, 2.8]	NA	0.89 (2.1) [−0.60, 2.4] [0.076, 1.7]	NA	NA
Worst pain in the past 24 h	5.3 (2.5) [3.6, 6.9] [4.4, 6.2]	3.7 (2.1) [2.3, 5.1] [3, 4.5]	3.8 (3.0) [1.9, 5.8] [2.8, 4.9]	1.5 (2.1) [0.16, 2.9] [0.78, 2.3]	1.5 (2.6) [−0.28, 3.2] [0.50, 2.4]	−0.091 (2.3) [−1.6, 1.5] [−0.94, 0.76]	5.7 (1.9) [4.3, 7.1] [5, 6.4]	4.1 (2.5) [2.3, 5.9] [3.1, 5.1]	4.0 (2.6) [2.2, 5.8] [3, 5]	1.6 (1.8) [0.33, 2.9] [0.91, 2.3]	1.7 (1.5) [0.63, 2.8] [1.1, 2.3]	0.10 (1.7) [−1.1, 1.3] [−0.57, 0.77]
Worst pain in the past 4 weeks	6.3 (2.7) [4.4, 8.1] [5.3, 7.3]	4.9 (2.6) [3.1, 6.7] [3.9, 5.9]	NA	1.4 (2.3) [−0.21, 2.9] [0.50, 2.2]	NA	NA	6.2 (1.3) [5.3, 7.1] 5.7, 6.7]	5.8 (2.5) [4.0, 7.6] [4.8, 6.8]	NA	0.40 (2.2) [−1.2, 2.0] [−0.44, 1.2]	NA	NA
Least pain in the past 24 weeks	1.9 (2.2) [0.45, 3.4] [1.1, 2.7]	1.7 (1.6) [0.64, 2.8] [1.1, 2.3]	1.4 (1.9) [0.11, 2.6] [0.68, 2]	0.18 (2) [−1.2, 1.5] [−0.55, 0.91]	0.55 (1.6) [−0.55, 1.6] [−0.06, 1.1]	0.36 (1.2) [−0.45, 1.2] [−0.08, 0.81]	2.0 (1.8) [0.69, 3.3] [1.3, 2.7]	1.1 (1.3) [0.18, 2.0] [0.60, 1.6]	1.1 (1.5) [0.01, 2.2] [0.51, 1.7]	0.90 (1.1) [0.11, 1.7] [0.47, 1.3]	0.90 (1.4) [−0.14, 1.9] [0.34, 1.5]	0.0 (0.47) [−0.34, 0.34] [−0.18, 0.18]
Least pain in the past 4 weeks	2.1 (2.6) [0.33, 3.9] [1.1, 3.1]	1.2 (1.4) [0.24, 2.1] [0.67, 1.7]	NA	0.91 (1.9) [−0.38, 2.2] [0.20, 1.6]	NA	NA	1.5 (2.2) [−0.09, 3.1] [0.64, 2.4]	1.6 (2.0) [0.20, 3.0] [0.84, 2.4]	NA	−0.10 (0.88) [−0.73, 0.53] [−0.44, 0.24]	NA	NA
Average pain in the past 24 h	3.6 (2.0) [2.3, 5.0] [2.9, 4.4]	2.4 (1.9) [1.1, 3.6] [1.7, 3.1]	2.5 (2.1) [1.1, 4.0] [1.8, 3.3]	1.3 (1.7) [0.11, 2.4] [0.63, 1.9]	1.1 (1.4) [0.17, 2.0] [0.58, 1.6]	−0.18 (0.87) [−0.77, 0.41] [−0.50, 0.14]	3.6 (1.4) [2.6, 4.6] [3, 4.2]	2.8 (2.0) [1.3, 4.3] [2, 3.6]	2.9 (2.0) [1.5, 4.3] [2.1, 3.7]	0.80 (1.9) [−0.54, 2.1] [0.07, 1.5]	0.70 (1.5) [−0.37, 1.8] [0.12, 1.3]	−0.10 (0.99) [−0.81, 0.61] [−0.49, 0.29]
Average pain in the past 4 weeks	4.0 (2.0) [2.6, 5.4] [3.2, 4.8]	2.6 (2.2) [1.2, 4.1] [1.8, 3.4]	NA	1.4 (1.6) [0.31, 2.4] [0.79, 1.9]	NA	NA	3.9 (1.7) [2.7, 5.1] [3.3, 4.5]	3.0 (1.9) [1.7, 4.3] [2.3, 3.7]	NA	0.90 (1.5) [−0.19, 2.0] [0.31, 1.5]	NA	NA
Current pain (at the time of assessment)	2.7 (1.8) [1.5, 3.9] [2.1, 3.4]	2.2 (2.0) [0.84, 3.5] [1.4, 2.9]	1.8 (2.0) [0.62, 3.4] [1.2, 2.8]	0.55 (2) [−0.78, 1.9] [−0.18, 1.3]	0.73 (2) [−0.62, 2.1] [−0.01, 1.5]	0.18 (1.3) [−0.66, 1.0] [−0.28, 0.64]	2.4 (1.5) [1.3, 3.5] [1.8, 3]	1.8 (1.8) [0.50, 3.1] [1.1, 2.5]	2.3 (2.4) [0.58, 4.0] [1.4, 3.2]	0.60 (1.9) [−0.76, 2.0] [−0.14, 1.3]	0.10 (1.8) [−1.2, 1.4] [−0.60, 80]	−0.50 (2.5) [−2.3, 1.3] [−1.5, 0.47]
**Pain unpleasantness, *M (SD)***	6.5 (2.8) [4.7, 8.4] [5.5, 7.6]	3.9 (3.0) [1.9, 5.9] [2.8, 5]	3.6 (2.9) [1.7, 5.6] [2.6, 4.7]	2.6 (3.7) [0.17, 5.1] [1.3, 4]	2.9 (2.9) [0.95, 4.9] [1.8, 4]	0.27 (2.1) [−1.1, 1.7] [−0.48, 1]	7.1 (3.4) [4.7, 9.5] [5.8, 8.4]	4.3 (2.5) [2.5, 6.1] [3.3, 5.3]	5.3 (3.3) [3.0, 7.6] [4, 6.6]	2.8 (3) [0.62, 5.0] [1.6, 4]	1.8 (3.6) [−0.81, 4.4] [0.38, 3.2]	−1.0 (2.2) [−2.6, 0.58] [−1.9, −0.14]
**Pain bothersomeness, n (%),** No bothersome	9 (81.8%)	10 (90.1%)	10 (90.1%)	NA	NA	NA	7 (70%)	9 (90%)	10 (100%)	NA	NA	NA
**Physical function, physical activity, and participation-KOOS, *M (SD) and 95% CI—*** *(higher the score, higher function)*												
KOOS pain	56 (14) [47, 66] [51, 62]	59 (18) [47, 72] [53, 66]	NA	3.1 (17) [−8.3, 14] [−3.2, 9.3]	NA	NA	51 (15) [40, 62] [45, 57]	60 (16) [49, 72] [54, 67]	NA	9.2 (12) [0.51, 18] [4.5, 14]	NA	NA
KOOS symptom	40 (13) [31, 49] [35, 45]	44 (10) [37, 51] [40, 47]	NA	3.2 (9.3) [−3.1, 9.5] [−0.23, 6.6]	NA	NA	47 (13) [38, 57] [42, 53]	51 (12) [42, 59] [46, 55]	NA	3.3 (14) [−6.8, 13] [−2.2, 8.8]	NA	NA
KOOS ADL	68 (13) [59, 77] [63, 73]	68 (16) [57, 79] [62, 74]	NA	−0.36 (10) [−7.2, 6.5] [−4.1, 3.4]	NA	NA	68 (17) [56, 80] [62, 75]	64 (19) [50, 78] [57, 71]	NA	−4.1 (17) [16, 8.3] [−11, 2.6]	NA	NA
KOOS sports/recreation	34 (27) [14, 53] [23, 44]	53 (28) [33, 73] [42, 64]	NA	18 (33) [−4.1, 40] [5.8, 30]	NA	NA	45 (28) [25, 65] [34, 56]	44 (20) [29, 58] [36, 51]	NA	−1.5 (26) [−20, 17] [−12, 8.8]	NA	NA
KOOS QOL	42 (15) [32, 52] [37, 48]	49 (12) [41, 57] [44, 54]	NA	6.8 (12) [−1.5, 15] [2.2, 11]	NA	NA	45 (11) [37, 53] [41, 49]	46 (10) [39, 54] [42, 50]	NA	1.3 (8.9) [−5.0, 7.6] [−2.1, 4.7]	NA	NA
KOOS aggregate	47 (12) [39, 56] [43, 52]	54 (15) [44, 64] [48, 59]	NA	6.2 (13) [−2.6, 15] [1.4, 11]	NA	NA	51 (12) [43, 60] [47, 56]	53 (11) [45, 61] [49, 57]	NA	1.6 (12) [−6.8, 10] [−3, 6.2]	NA	NA

**TABLE 5:** Pain and interference measures during NF training sessions.

Domains and variables	Active group (*n* = 11)			Sham group (*n* = 10)		
	Week 1	Week 2	Week 3	Week 1	Week 2	Week 3
**Pain severity-BPI, *M (SD)***						
Pain severity sub-score	2.7 (1.4)	2.2 (1.6)	2.0 (2.0)	3.2 (1.2)	2.9 (1.7)	2.5 (1.9)
Worst pain in the past 24 h	4.3 (2.1)	3.5 (2.4)	3.3 (2.9)	4.9 (2.0)	4.4 (2.4)	3.7 (2.7)
Least pain in the past 24 weeks	1.5 (1.5)	1.3 (1.5)	1.5 (2.0)	1.8 (1.2)	1.2 (1.1)	1.6 (1.2)
Average pain in the past 24 h	2.9 (1.5)	2.3 (1.7)	2 (1.9)	3.1 (1.3)	3.1 (1.9)	2.5 (2.2)
Current pain (at the time of assessment)	2.2 (1.3)	1.8 (1.7)	1.8 (1.8)	2.9 (1.4)	3 (2.2)	2.3 (1.8)
**Pain interference-BPI, single item, *M (SD)***	1.9 (1.8)	1.8 (1.8)	1.4 (2.4)	2.4 (2.0)	2.4 (2.3)	2.3 (2.5)
**Pain unpleasantness, *M (SD)***	4.4 (2.0)	3.9 (2.8)	3.6 (3.4)	6.3 (3.2)	5.6 (3.3)	4.5 (3.3)

**TABLE 6:** Experimental pain measures and physical performance measures.

Domains and variables	Active group (*n* = 11)		Sham group (*n* = 10)	
	Baseline	Post-intervention	Baseline	Post-intervention
**Quantitative sensory testing measures**				
**Vibration detection threshold**				
Symptomatic score/8	4.5 (1.4)	4.2 (1)	4.9 (1.6)	4.6 (1.5)
Asymptomatic score/8	4.4 (1.1)	4 (1)	5.2 (1.5)	4.7 (1.3)
**Mechanical temporal summation**				
MTS-Symptomatic VAS change scores	16.8 (18.4)	15.8 (17.3)	7.9 (9.5)	13.6 (20)
MTS-Asymptomatic VAS change scores	11.2 (10.6)	14.9 (17.3)	6.3 (11.8)	12.6 (16.6)
MTS-Dorsal wrist VAS change scores	5.3 (9.8)	6.2 (5.3)	3.8 (9.3)	4.2 (6.7)
MTS-Tibialis anterior VAS change scores	12.4 (13.5)	17.7 (22)	7.1 (11.9)	11.7 (14.2)
**Pressure pain threshold, *M* (*SD*)**				
PPT-Symptomatic (kPa)	232.9 (123.8)	254.8 (135.2)	291.9 (219.6)	354.7 (238.1)
PPT-Asymptomatic (kPa)	228.6 (103.6)	216 (104.8)	308.4 (213.5)	352.1 (261.9)
PPT-Dorsal wrist (kPa)	289.7 (170.3)	256.1 (115.6)	281.8 (137.6)	515.2 (673.1)
PPT-Tibialis anterior (kPa)	270.9 (96.3)	242.3 (116.6)	301.5 (181.8)	318.3 (159.2)
PPT-Thumb nail (kPa)	227.9 (71.3)	213.3 (89.3)	285.5 (167.6)	300.9 (192)
**Cold hyperalgesia, *M* (*SD*)**				
PVAS-Symptomatic	52.3 (28.6)	35.2 (16.8)	32.7 (19.1)	27.7 (22.8)
PVAS-Asymptomatic	48.6 (29.6)	34.5 (14.8)	31.2 (18.8)	28.9 (20.6)
**Conditioned pain modulation, *M* (*SD*)**				
CPM30 s % change scores (PPT-P4 at 30 s—PPT-p4 preconditioning score)	14 (40.1)	13.9 (17.1)	13.2 (28.4)	27.4 (32.2)
CPM60 s % change scores (PPT-P4 at 60 s—PPT-p4 preconditioning score)	20.3 (41.4)	6.8 (21.5)	−14.5 (21.7)	25.1 (31)
CPM90 s % change scores (PPT-P4 at 90 s—PPT-p4 preconditioning score)	22.7 (40.9)	7.4 (11.5)	−0.8 (21)	17.9 (26.1)
**Tactile acuity and motor imagery performance**				
**Body schema integrity, *M* (*SD*)**				
Time (seconds)				
Left	2.1 (0.5)	2.3 (0.4)	2.4 (0.4)	2.1 (0.3)
Right	2.4 (0.6)	2.3 (0.4)	2.3 (0.4)	2.2 (0.4)
Accuracy (percentage)				
Left	65.8 (11.7)	74.2 (9.6)	71.3 (16.6)	70.3 (10.9)
Right	72.7 (13.2)	73 (9.2)	70 (15.3)	72.7 (8.3)
**Two-point discrimination (cm), *M* (*SD*)**				
Symptomatic	4.7 (2)	3.8 (1.3)	5.5 (2)	5.1 (1.3)
Asymptomatic	4.2 (1.8)	3.6 (1)	3.8 (1.8)	4.4 (1.3)
**Sensitivity to physical activity**				
Discomfort (Peak discomfort-baseline)	3.6 (2.5)	2.9 (2.6)	2.4 (2.3)	1.7 (1.8)
Distance in meters	454.3 (131.4)	425.5 (131.2)	446 (60.3)	445.4 (116.9)
**Physical performamce-30 s chair stand, *M* (*SD*)**	9.5 (2.7)	10.5 (3.4)	11.1 (2.7)	12.7 (3.6)

**FIGURE 6:**
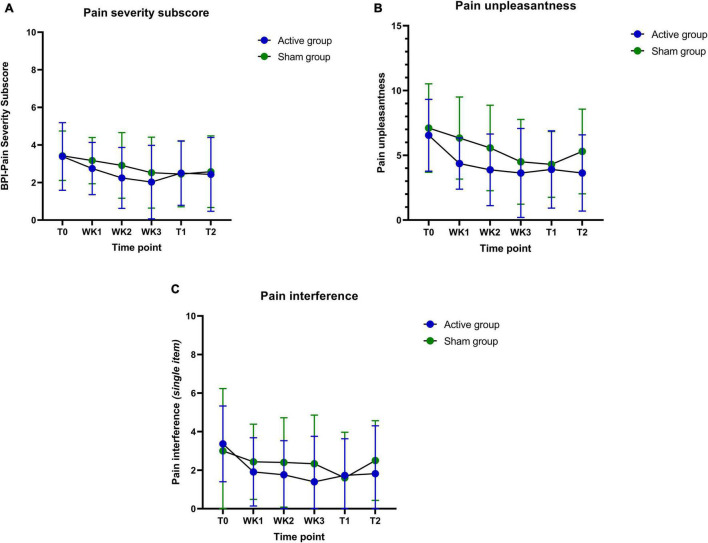
**(A–C)** Pain severity, pain unpleasantness, and pain interference (baseline: T0; weekly: WK1-WK3; post-intervention: T1 and the follow-up: T2) measures during the training period.

Another observation includes the improvements in pain severity during immediate and follow-up assessments when compared to baseline. The sham group tends to taper off on pain severity after the training period, whereas the active training group effect is either maintained or further improved over time (Figures 6A–C). Also, participants in the active ISF-NF group have shown a consistent reduction in their pain interference score during their NF sessions. In contrast, sham ISF-NF has not demonstrated a reduction in the pain interference scores.

### EEG based outcome measures

#### Region of interest analysis

**FIGURE 7:**
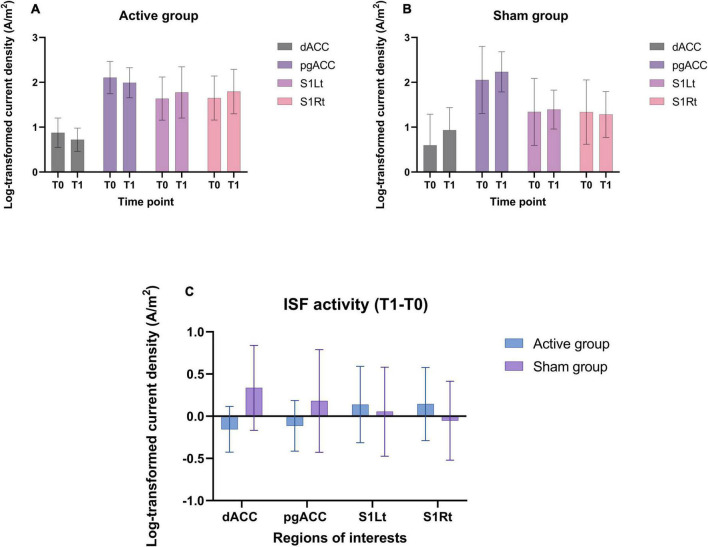
**(A,B)** Shows the CSD (mean and standard deviation) of ISF band in different ROIs utilized in this trial for both groups. **(C)** The mean difference (T1-T0) of CSD of ISF band at the ROIs for both the groups.

The log transformed CSD values were averaged across all voxels belonging to the dACC, pgACC, S1Rt, and S1Lt for the ISF band (Figures 7A–C).

**FIGURE 8:**
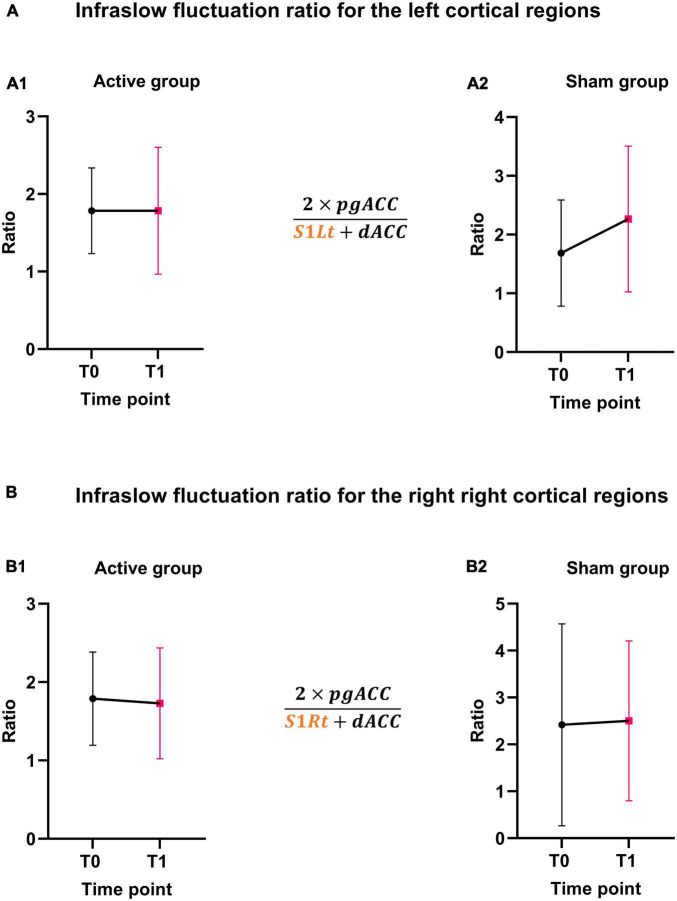
ISF ratio changes. **(A,B)** Shows the ISF-NF ratio changes before and after NF training for the active and sham groups for left and right cortical regions.

The balance between the areas of interest (pgACC, dACC, and SSC) for the ISF band was calculated and illustrated in Figures 8A,B.

#### Functional connectivity measure

**FIGURE 9:**
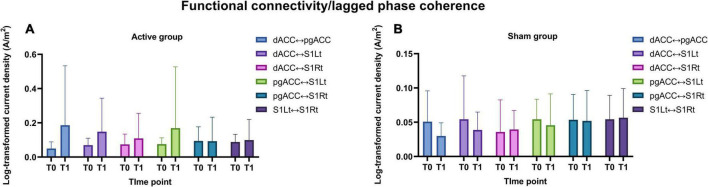
**(A,B)** Illustrates the functional connectivity changes (means and standard deviations) for the ISF band frequency between the ROIs for both active and sham group.

The strength of the ISF band FC between the four ROIs for both the groups are represented in Figures 9A,B.

## Discussion

### Principal findings

To our knowledge, this study is the first to assess the feasibility, safety, and acceptability of ISF-NF training in a musculoskeletal pain population using a randomized, double-blinded, sham-controlled design. The trial results suggest that a future fully powered clinical trial to evaluate the effectiveness of ISF-NF in KOA is feasible, safe, and acceptable, with participants rating high levels of perceived effectiveness. The overall willingness to participate in the trial was high. The results demonstrate a 100% treatment fidelity with all the NF training components, including dosage, duration, and frequency of intervention was delivered and completed as per the study protocol (Mowbray et al., 2003; Kubiak et al., 2014). The study also maintained a 95.4% retention rate, with participants in both groups had reported higher motivation and engagement levels with the NF training sessions. Both the planned primary and secondary outcome measures were feasible and acceptable. Participants in both active and sham groups reported no serious adverse events.

### Recruitment and COVID related challenges

As planned, we recruited the desired number of participants in 5 months of the recruitment period (Mathew et al., 2020). While the COVID-19 pandemic and associated lockdown periods impacted the study recruitment strategies, this extended the overall study recruitment and data collection period. Most of the study participants were recruited through newspaper advertisement (82%), and the rest of them were recruited through social media (9%) and university email circulation (9%). Local community newspaper advertisement is considered the most strategic recruitment method for future trials for the defined age limits.

### Diversity of participants

The majority of the participants were New Zealand European and middle-aged females, consistent with the previous NZ-based studies on KOA (Abbott et al., 2017; Awatere, 2018; Wilson and Abbott, 2019). However, recruitment of a more heterogeneous ethnic sample, including Māori and Pacifica populations with equal sex representation, is warranted in future research. Participants in the active group had an average pain duration of 5 years, and the sham group with nearly 3 years. However, the average pain reported by both the groups in the last 3 months was comparable at baseline (6/10), and both groups displayed a similar number of co-morbidities.

### Acceptability and perceived levels of effectiveness of the intervention

Acceptability and perceived levels of effectiveness are two important domains in the design, evaluation, and implementation of health care interventions (Sekhon et al., 2017; Suka et al., 2017). Our results show that participants in both groups rated high levels of acceptance. Low acceptability and negative treatment perception could influence treatment adherence and outcomes (Milosevic et al., 2015).

Participants in both groups also reported moderate to high levels of perceived effectiveness of the training. The domain is understudied in health care interventions, and it is important to conduct standalone research or incorporate it to add further value to future clinical trials. In the current study, we have used only a single-item measure to rate the level of the acceptability and perceived levels of effectiveness with NF training. In the future, studies need to consider incorporating a qualitative methodology approach to investigate participants’ perception of the NF training to understand the depth of the perspective component associated with the intervention.

Participants did not report difficulties or concerns with the assessments, and training procedures, and follow-up. Although all participants completed their training sessions, some of the training sessions were rescheduled within the same week. The main reason for rescheduling is participants’ unavailability to undergo training. Another challenge was that participants were not comfortable returning to work/home with dried electrode gel. Although participants were offered the option to wash their hair at the study location, none did so due to other commitments/personal reasons following the training session. Future research could consider incorporating dry electrodes for training (Yu et al., 2016; Pei et al., 2018).

### Adverse events

Evidence from previous studies has reported various side effects associated with NF training (Lubar and Shouse, 1976; Hammond and Kirk, 2008; Todder et al., 2010; Lansbergen et al., 2011; Dongen-Boomsma, 2014; Rogel et al., 2015). It appears that different NF protocols are associated with a varying set of side effects and cannot be generalized. However, all these studies used different NF training protocols, and none of them was designed to modulate the ISF band. To our knowledge, this is the first clinical trial performed a structured methodology using a full DESS scale to document the adverse effects associated with ISF-NF protocol training. In the absence of any serious adverse events, participants in both groups have reported improvement with their pre-existing symptoms during NF training. The results support our previous study on ISF-NF training in food addiction, which also reported no serious/persisting side effects associated with ISF-NF training (Leong et al., 2018).

### Factors influencing the level of engagement with training

The level of motivation and mood may impact the participants’ engagement with the NF training (Kleih-Dahms et al., 2011; Ros et al., 2020). Recently NF studies have established the importance of motivational value as an essential addition in predicting the success of NF training (Diaz Hernandez et al., 2018; Perez-Elvira et al., 2021). Our study adds to the growing literature regarding the predictors and factors influencing NF training outcomes (Weber et al., 2020). In our study, participants in both groups were highly motivated and engaged in undergoing the NF training. Our findings support the notion that the individual’s daily mood and motivation will significantly impact the training outcome. It would be of value if the future trial could investigate the relationship between the everyday mood and motivation with the NF training EEG measures. Also, the training time of the day is an important factor determining the participant’s engagement with NF training. Participants who opted for afternoon training sessions displayed higher slowing of the alpha rhythm and enhancement of the theta power indicative of drowsiness during the training time (Noreika et al., 2020). It is important to monitor the participant’s alertness during the training time and need to provide verbal commands if necessary to improve the alertness and engagement with the training sessions. Participants should be fully relaxed and involved with the NF training to learn the patterns of “reward” and maintain the percent of success during the training. However, this needs to be considered an objective to predict the NF outcome in future clinical trials.

### Blinding integrity

We found that most of the active ISF-NF participants could predict their group allocation correctly. A potential reason could be due to reduction of their symptoms. On the other hand, most sham group participants indicated that they were in the active group, as they believed and were hopeful of being in it. This could potentially contribute to the non-specific effect observed in the sham group and needs further investigation. Future trials must incorporate methodologies to investigate any relationship between the subjective pain measures observed in the sham group with their associated motivational or belief neural network (Taylor et al., 2004; Wiech et al., 2008; Zhu et al., 2012; Sterpenich et al., 2014). The failure to identify the group allocation also implies the integrity of the sham methodology used in this trial. However, this does not entirely omit the therapeutic element (Brim and Miller, 2013; Pigott et al., 2018). Also, future trials must evaluate the blinding integrity of the assessor to eliminate the potential bias in the QST measures.

### Changes in the clinical outcomes

This study demonstrated changes in the clinical outcomes in both active and sham ISF-NF groups. It is noteworthy that the active ISF-NF group showed greater improvements in pain unpleasantness and pain interference scores. This study also observed a group difference in the time course effect on the symptoms during training, post-training (immediate and follow-up) assessments. The time course effect of NF is discussed in previous literature (Rance et al., 2018), and the same trend has been observed in previous NF clinical trials (Van Doren et al., 2019; Arnold et al., 2020). Considering the persistence and continuing improvement in symptoms after NF treatment highlights the importance of including regular longer follow-up assessment sessions in future trials to ensure that the time point of greatest effect is sampled (Rance et al., 2018).

Considering the nature of the study design, we haven’t performed any statistical analysis for the EEG changes. However, the descriptive EEG data will help design and perform sample size calculations for future studies investigating the efficacy of ISF-NF training inducing neural changes in the chronic pain population. Future trials also need to consider a full band activity (>0.1 Hz) and connectivity EEG analysis to explore the effects of ISF-NF and its relationship with change in clinical pain outcomes. The results also demonstrated a positive trend in the balance between the ascending (SSC and dACC) and descending (pgACC) pain pathways in the active group. Moreover, the findings support the concept of the brain imbalance model of chronic pain (Vanneste et al., 2018; De Ridder et al., 2021; Vanneste and De Ridder, 2021), and thus, the imbalance can potentially be improved using ISF-NF intervention. However, these results need to be tested on a larger population to determine the changes in ratio/balance between the pain pathways and their association with the difference in the clinical and experimental pain outcomes following NF training.

### Limitations and recommendations

Our findings should be interpreted with consideration of the study’s limitations. We recruited a small sample of KOA individuals with a homogeneous sex and race distribution, which restricts the generalizability of the study findings. The feasibility of long-term follow-up assessment and a higher number of NF training sessions was not studied in this trial. Despite the limitations, this study provides an important foundation for designing and implementing future clinical trials investigating ISF-NF protocols. Our study outcomes regarding the feasibility, safety, and acceptability provide validation for such future studies to replicate and explore the effectiveness of ISF-NF for chronic musculoskeletal pain conditions. We recommend a fully powered RCT to investigate the ISF-NF balance training protocol’s effectiveness with a justifiable sample size calculated based on the reported means and standard deviations for detecting differences between groups of at least medium effect size (Cohen’s d = 0.5) with 80% statistical power.

## Conclusion

In conclusion, this feasibility clinical trial suggests that a fully powered clinical trial is feasible and safe to be conducted in people with KOA. Moreover, people with KOA reported high levels of acceptability and perceived high levels of effectiveness of training and higher levels of motivation and engagement with the NF training. The study provides variability in the clinical and EEG measures, thus informing the future studies in ISF-NF for the estimation of sample size for a fully powered clinical trial.

## Data availability statement

The datasets generated during and/or analyzed during the current study are available from the corresponding author on reasonable request.

## Ethics statement

The studies involving human participants were reviewed and approved by Health and Disability Ethics Committee (HDEC), New Zealand (19CEN182), and the cultural consultation was obtained from the Ngāi Tahu Research Consultation Committee (5733_21392). The patients/participants provided their written informed consent to participate in this study.

## Author contributions

DD, DA, RM, and JM provided the cortical areas to be targeted, their coordinates, and the desired training protocol. MS developed the ISF-NF programme based on the provided regions and coordinates. JM conducted the participant recruitment, data collection, EEG data processing, analysis, and wrote the first draft. All authors contributed to the interpretation of the results, manuscript revision and reviewed the final version making the necessary changes, approved the submitted version, and agreed to be accountable for the content of the work.

## Footnotes

1
www.neurosynth.org
