# Supplementary material for: Source localized infraslow neurofeedback training in people with chronic painful knee osteoarthritis: A randomized, double-blind, sham-controlled feasibility clinical trial
Source: Front Neurosci. 2022 Jul 28;16:899772. doi: 10.3389/fnins.2022.899772 (PMC9366917; doi:10.3389/fnins.2022.899772)
Supplement: Supplementary file 1 [file Table_1.DOCX]

| **Supplementary table 1: Summary of the outcome measures** | | | | |
| --- | --- | --- | --- | --- |
| **Questionnaires/measures** | **Components/Dimensions/Items** | **Scoring criteria/interpretation** | | **Time point** |
| **Primary outcome piloted for the full trial** | | | | |
| Brief Pain Inventory (BPI) *(Keller et al., 2004; Mendoza et al., 2006)* | 24 hr and 4 weeks Pain severity items (worst pain, average pain, least pain, and current pain). | 0-‘No pain’ to 10-‘Pain as bad as you can imagine’ | | 24 hr items: S1 – S12  4-week item: S1 and S11 |
|  | Mean pain severity sub-score | Composite item taking the mean of the four 24hr severity items | | S1 – S12 |
|  | Seven interference items (how paininterferes with activity, mood, relations with others, walking ability, work, enjoyment of life, and sleep) | 0-‘does not interfere’ to 10-‘completely interferes’ | | Full scale: S1 and S11  Single item (component A): S2 – S10 |
| VAS-unpleasantness scale (Price et al., 1983; Harkins et al., 1989; Starr et al., 2011) | Pain unpleasantness in the last 24-hours | 0-being ‘not at all pleasant’ and 15-being ‘most unpleasant imaginable’ | | S1 – S12 |
| Pain bothersomeness scale (Dunn and Croft, 2005), (Thomas et al., 2019) | Pain bothersomeness in the last 24-hours | Five possible responses: “not at all”, “slightly”, “moderately”, “very much”, and “extremely”.  Bothersomeness was dichotomized into bothersome (i.e., reports of “very much” or “extremely” bothersome) and not bothersome (i.e., reports of “moderately,” “slightly,” or “not at all” bothersome). | | S1 – S12 |
|  | Pain bothersomeness in the last week |  |  |  |
| Knee injury and Osteoarthritis Outcome Score (KOOS) *(Hawker et al., 2008; Peer and Lane, 2013)* | 42-item self-reported questionnaire.  5 reported dimensions: Pain (9 items), other symptoms (7 items), function in daily living (17 items), function in sport and recreation (5 items), and knee-related quality of life (4 items) | 5-point Likert scale, with anchors of zero (no problems) to 4 (extreme problems). Scores are transformed to a 0-to100 scale, with zero representing extreme knee problems and 100 representing no knee problems | | S1 and S11 |
| **Secondary outcomes piloted for the full trial** | | | | |
| PainDETECT questionnaire (PD-Q) *(Hochman et al., 2011; Hochman et al., 2013)*. | Nine items (seven evaluating pain quality, one evaluating pain pattern, and one evaluating pain radiation) | Response: Never (0), Hardly noticed (1), Slightly (2), Moderately (3), Strongly (4), and Very strongly (5)  Total score/38. ≥19 indicative of a likely neuropathic pain, ≤12: nociceptive pain, and 13–18: possible neuropathic pain component [or mixed type]. | | S1 and S11 |
| International Physical Activity Questionnaire—short form (IPAQ-SF) (Maddison et al., 2007) | 9 items on the time spent doing walking, moderate- to vigorous-intensity activities, and sedentary activities. | Both categorical (low, moderate, and high based on PA recommendations) and continuous variables (walking MET-min/wk, moderate MET-min/wk, vigorous MET-min/wk, total PA MET-min/wk, total activity min/wk, and total days of activity) were calculated for scoring the IPAQ-SF (Gay et al., 2019) | |  |
| Sedentary Behaviour Questionnaire (SBQ) (Rosenberg et al., 2010; Mani et al., 2019) | 9 behaviours for weekdays and weekends separately (watching television, playing computer/video games, sitting while listening to music, sitting and talking on the phone, doing paperwork or office work, sitting and reading, playing a musical instrument, doing arts and crafts, sitting and driving/riding in a car, bus, or train) | Response: “none” to “6 hours or more”  Mean duration (hours per day) spent on a typical weekday and weekend day was computed.  A weighted daily estimate of sedentary time (hours per day) was calculated as *[(∑(sedentary time during a typical weekday) (∑ (sedentary time during a typical weekend day)/7* | |  |
| Social support and Pain Questionnaire (SPQ) (Van Der Lugt et al., 2012; He and Wang, 2017) | 6-item tool was used to evaluate the participant’s satisfaction with pain-related social support | Likert scale from 0 to 4 (0 = very dissatisfied, 1 = dissatisfied, 2 = neutral, 3 = satisfied, 4 = very satisfied)  Higher scores indicate more satisfaction. | |  |
| Lubben Social Network Scale (Musich et al., 2019) (Gray et al., 2016) | 6 item tool that measures social networks through relationships with family and friends | Graded from 0 to 5 points with a total score of 0 to 30 points. A higher score indicating more social engagement. | |  |
| Keele Assessment of Participation (KAP) (Wilkie et al., 2005; Wilkie et al., 2011) | 11-item measures mobility, self-care, domestic life, and interpersonal interaction | 5-point scale (all of the time, most of the time, some of the time, a little of the time, none of the time).  Dichotomized to define the presence (some, a little, none of the time) or absence (all or most of the time) of participation restriction | |  |
| EuroQol EQ-5D (Oppe et al., 2007; Bilbao et al., 2018) | Dimensions: mobility, self-care, usual activities, pain/discomfort, and anxiety/depression | Categorical scale: 1-no problems, 2-slight problems, 3-moderate problems, 4-severe problems, and 5-extreme problems. The state is defined by a five-digit code. | |  |
| EQ-VAS scale | Overall health status | 0-‘the worst health you can imagine’ and 100-‘the best health you can imagine’ | |  |
| Short Form Health Survey (SF-36) (McHorney et al., 1994; Webster and Feller, 2016) | Used only four key components (overall general health, Compared to one-year, general heath now, paininterfered with normal work in the past 4 weeks, and Physical health/emotional problems interfered with social activities in the past 4 weeks) | Categorical scale: 1-Excellent, 2-Very Good, 3-Good, 4-Fair, and 5-Poor. A higher score indicates a higher impact on the quality of life. | |  |
| Pittsburgh Sleep Quality Index (PSQI) (Omachi, 2011) | 7 components: subjective sleep quality (1 item), sleep latency (2 items), sleep duration (1 item), habitual sleep efficiency (3 items), sleep disturbances (9 items), use of sleeping medications (1 item), and daytime dysfunction (2 items) | Response options vary with different items.  overall score range is 0 –21 points, with higher scores worse sleep quality | |  |
| Knee Osteoarthritis Fears and Beliefs Questionnaire (KOFBeQ) (Benhamou et al., 2013) | An 11-item questionnaire assessing fears and beliefs of individuals with KOA | 10-point Likert scale (0-totally agree and 9- totally disagree). Higher scores indicate substantial fears and beliefs. | |  |
| Pain vigilance and awareness questionnaire (PVAQ) *(Roelofs et al., 2003; Herbert et al., 2014)* | 16 items measure the frequency of habitual “attention to pain” over the past 2 weeks | 6-point Likert scale (0-never to 5-always), and the total score ranges from 0 to 80. Higher scores indicate greater levels of pain vigilance and awareness | |  |
| Pain Catastrophizing Scale (PCS) (Severeijns et al., 2001)*,* (Ong et al., 2021) | 13 items that measures three dimensions of catastrophizing: rumination (sum of items 8, 9, 10, 11), magnification (sum of items 6, 7, 13), and helplessness (sum of items 1, 2, 3, 4, 5, 12) | 5-point Likert scale (0-not at all to 4-all the time). Total score ranges from 0 to 52, where higher scores indicate greater levels of catastrophic thoughts about pain. | |  |
| Pain Self-Efficacy Questionnaire (PSEQ-2) (Nicholas, 2007; Nicholas et al., 2015) | A two-item scale was used to rate the confidence of the participant. | 7-point scale, with 0 = not at all confident and 6 = completely confident. A score ≥8 reflects a desirable level of pain self-efficacy or confidence in functioning in the presence of pain. | |  |
| Brief Resilience Scale (BRS) (Smith et al., 2008; Bartley et al., 2019) | Six-item tool to measure one’s ability to bounce back from stress. | Five-point Likert scale: 1-strongly disagree, 2-disagree, 3-neutral, 4-agree, 5-strongly agree. The final score is derived by dividing the total score by the total number of questions answered.  BRS score: 1.00 - 2.99 is low resilience; 3.00-4.30 is normal resilience, and 4.31-5.00 is high resilience. | |  |
| Coping Strategies Questionnaire (CSQ) (Alschuler et al., 2013) | The 14-item scale used to score seven pain coping strategies; adaptive strategies: Diverting Attention, Reinterpreting Pain Sensations, Ignoring Sensations, Coping Self-Statements, Increased Behavioral Activities; maladaptive strategies: Catastrophizing, Praying and Hoping | 0 to 6 scale (0-never do, 3- sometimes do that and 6-always do that).  A higher score indicates an increased frequency of use of each pain-coping response | |  |
| Chronic Pain Acceptance Questionnaire (CPAQ) (Fish et al., 2010; Baranoff et al., 2014) | Eight-item version to assess two related behavioral processes; activity engagement and pain willingness. | 7-point Likert scale (0-never true, and 6-always true). A higher score indicates higher acceptance. | |  |
| Depression, Anxiety, and Stress Scale (DASS-21) (Wood et al., 2010; Youngcharoen et al., 2017; Uritani et al., 2020) | 21-item was used to measure three psychological constructs: depression, anxiety, and stress over the past week. | 4 point Likert scale; 0-Did not apply to me at all,  1-Applied to me to some degree, or some of the time,  2-Applied to me to a considerable degree or a good part of the time, and 3- Applied to me very much or most of the time. A higher score indicating higher levels of depression, anxiety, and stress. | |  |
| Central Sensitisation Inventory (CSI)  (Mayer et al., 2012)*-*(Neblett et al., 2015; Lluch Girbés et al., 2016; Gervais-Hupé et al., 2018) | 25 health-related symptoms common to central sensitivity syndromes. | Five point Likert scale: Never = 0, Rarely = 1, Sometimes = 2, Often = 3, Always = 4.  Higher scores are associated with higher levels of self-reported symptoms. | |  |
| Questionnaire for Current Motivation-Brain Commuter Interference (QCM-BCI) (Rheinberg et al., 2001; Nijboer et al., 2010) | 18 statements that assess four different components of motivation: (1) mastery confidence, (2) fear of incompetence (3) interest, and (4) challenge. | 7-point Likert scale; 1-strongly disagree to 7-fully agree. | | S1 |
| Visual Analogue Scale (VAS) Motivation (Kleih et al., 2011; Kleih and Kubler, 2013) | Participants were asked to indicate their motivation on a 10 cm long horizontal line (0- Not at all motivated? and 10-extremely motivated) | | | Before every NF session (S2 -S10) |
| Brief Mood Introspection Scale (BMIS) (Mayer and Gaschke, 1988; Kokkonen and Pulkkinen, 2001) | A single item tool was used to record the overall mood of the participant. | 11-point numeric scale, with 0 being very unpleasant and 10- being very pleasant. | | S2 to S10 |
| Level of Engagement (Ros et al., 2020) | 10-point Likert scale after every NF session, where 1 = least engaged and 10 = highly engaged | | | After every NF session (S2-S10) |
| **Experimental Pain Measures** | | | | |
| Pressure Pain Threshold (PPT) (Wylde et al., 2011; Mutlu and Ozdincler, 2015; Alahmari et al., 2020) | Locations: 1) 3 cm medial to the medial border of the patella, at the side of the knee joint line of the most painful knee, 2) 5 cm distal to the tibial tuberosity, over the tibialis anterior muscle ipsilateral to the most painful knee, 3) dorsum of the distal radioulnar joint of the contralateral forearm to the most painful knee joint, 4) base of the thumb nail of the contralateral upper limb (Moore et al., 2020) | | PPT was measured thrice on each site and the mean of three measurements was used for the analysis *(Nie et al., 2005; Riley et al., 2019)* | S1 and S11 |
| Mechanical Temporal Summation (MTS) *(Wylde et al., 2011; Neogi et al., 2015)* | TS was assessed using a nylon monofilament (Semmens monofilament 6.65, 300g)  Locations: medial joint line of the most painful knee and asymptomatic knee, the dorsum of the ipsilateral hand and 5 cm distal to the tibial tuberosity, over the tibialis anterior muscle ipsilateral to the most painful knee, in randomized order. *(Goodin et al., 2014; Mani et al., 2019)* | | Verbal 0-100 (NRS) rating of pain following a single contact and another 0-100 rating of their highest pain intensity experience following a series of 10 contacts.  MTS was calculated as the difference between the NRS rating after the first contact and the highest pain rating after the 10^th^ contact. |  |
| Conditioned Pain Modulation (CPM) *(Imai et al., 2016; Kennedy et al., 2016)* | Test stimulus: Suprathreshold PPT (Pain40) before (two trials) and after (at 30, 60, and 90 seconds).  Condition stimulus: immerse dominant hand in the water bath, maintaining the temperature at ~5±1°C, up to the wrist crease for 2 minutes. | | A percent change score was calculated for each time point (i.e., 30 seconds [CPM30sec], 60 seconds [CPM60sec], and 90 seconds [CPM90sec]) as below, with a positive score indicating an increase in PPTs (Pain40) after the conditioning stimulus and thus the presence of CPM effect (Mani et al., 2019).  𝐶𝑃𝑀 𝑝𝑒𝑟𝑐𝑒𝑛𝑡 𝑐ℎ𝑎𝑛𝑔𝑒 𝑠𝑐𝑜𝑟𝑒=Post score −Pre score/Pre score 𝑥 100.  Impaired CPM was defined as no change or a negative change in PPT measures, taken directly following the termination of the conditioning stimulus (cold pressor test) *(Lewis et al., 2012; Yarnitsky et al., 2015)* |  |
| Vibration detection threshold (VDT) (Jakorinne et al., 2018b) | Using a tuning fork (64 Hz Rydel-Seiffer tuning fork, 8/8 scale) placed on the medial tibial condyle with suprathreshold vibration intensity and kept there until the participant could no longer feel the vibration. (Panosyan et al., 2016) (Martina et al., 1998) | | A triangle and an arbitrary scale from 0 (minimum score) to 8 (maximum score). The vibration extinction threshold was considered as the nearest value to the apparent point of intersection of the virtual triangles when the subject indicated that vibration is no longer perceived. |  |
| Cold hyperalgesia (Tilley and Bisset, 2017) | Pain Visual Analogue Scale (PVAS) ice protocol by massaging the painful knee area with a 20 mm diameter ice cylinder for 10 seconds. | | Pain on a 100 mm pain n VAS, with 0 mm indicative of ‘no Pain at all’ and 100 mm indicative of the ‘worst Pain imaginable’. |  |
| Somatosensory reorganization and motor imagery performance | | | | |
| Tactile acuity (Jakorinne et al., 2018a) | Two-point discrimination threshold (TPD) was measured 2 cm medial of the medial border of the patella (using the tibiofemoral joint line as a reference point) (Stanton et al., 2013) | | TPD is the shortest distance between calliper points at which the participant could clearly detect two points instead of one. | S1 and S11 |
| Body part recognition task (Stanton et al., 2012; Stanton et al., 2013) | Performance accuracy on determining the left and right judgment (accuracy and reaction time) of the image (a body part) that appears on the iPad screen (Recognise software). | | Accuracy of the judgment was computed in percentage and was generated by the software (http://recognise.noigroup.com/recognise), with three trials |  |
| **Physical activity and performance measures** | | | | |
| Sensitivity to Physical Activity (SPA) (Vader et al., 2020) | A 6-minute walk (6MWT) test was performed to evaluate the level of knee discomfort. (Wideman et al., 2014) | | On a 0 (no discomfort) to 10 (extreme discomfort) numeric scale participants rated their discomfort 7 times once immediately before the task and once after each minute of walking.  SPA index was determined by calculating the difference between the peak knee discomfort during the 6MWT and baseline knee discomfort at rest immediately before the test. | S1 and S11 |
| Physical performance measure | 30-second chair stand test was performed for every participant (Dobson et al., 2013) | | A maximum number of chair stand repetitions possible in a 30 second period was noted (Suwit et al., 2020). |  |

| **Supplementary table 2: Changes in the secondary outcome measures** | | | | |
| --- | --- | --- | --- | --- |
| **Domains and variables** | **Active group (n = 11)** | | **Sham group (n = 10)** | |
|  | **Baseline** | **Post-intervention** | **Baseline** | **Post-intervention** |
|  | | | | |
| **PainDETECT total score**, ***M (SD)*** | 9 (3.7) | 8 (5.8) | 12 (5.8) | 10 (7.7) |
| Nociceptive pain (n [%]) | 9 (81.8%) | 8 (72.7%) | 5 (50%) | 6 (60%) |
| Mixed type (n [%]) | 2 (18.2%) | 3 (27.3%) | 4 (40) | 3 (30%) |
| Neuropathic pain (n [%]) | 0 | 0 | 1 (10%) | 1 (10%) |
| **Physical activity, Quality of life, Psycho-social, and sleep quality measures** | | | | |
| **Physical activity (IPAQ-SF), M (SD)** |  |  |  |  |
| Total days of activity (d/wk) | 6.6 (0.7) | 6.5 (1) | 6.7 (0.9) | 6.5 (1.3) |
| Total activity (min/wk) | 110 (72.2) | 102.3 (65.1) | 189.5 (143.6) | 222.5 (147.7) |
| MET-min/wk—vigorous | 640 (1143.2) | 545.5 (1141.4) | 1188 (1773.1) | 1248 (1813) |
| MET-min/wk—moderate | 1189.1 (1501.2) | 780 (844) | 1576 (1445) | 1902 (1513.2) |
| MET-min/wk—walking | 280.5 (244.2) | 442.5 (377.2) | 1011.5 (1271.3) | 924 (1038.4) |
| MET-min/wk—total | 2109.6 (1819.4) | 1768 (1348.4) | 3775.5 (4033.2) | 4074 (3409.4) |
| Physical activity categories (n [%]) |  |  |  |  |
| Low | 0 | 1 (9.1%) | 1 (10%) | 1 (10%) |
| Moderate | 8 (72.7%) | 7 (63.6%) | 3 (30%) | 3 (30%) |
| High | 3 (27.3%) | 3 (27.3%) | 6 (60%) | 6 (60%) |
| **Sedentary behaviour—daily estimates (h/d)** | 8.9 (3.1) | 9.3 (2.9) | 9.9 (4.3) | 8.5 (5.2) |
| **Social support and pain Questionnaire, M (SD)** | 17 3.3 | 18.3 3.4 | 15.2 5.3 | 16.4 5.5 |
| **Lubben Social Network Scale–6, M (SD)** | 15.6 7.3 | 17.2 6.3 | 17.9 7.3 | 17.1 5.5 |
| **Keele Assessment of Participation, n (%)** |  |  |  |  |
| Any restriction | 8 (72.7%) | 6 (54.5%) | 6 (60%) | 8 (80%) |
| No restriction | 3 (27.3%) | 5 (45.5%) | 4 (40%) | 2 (20%) |
| **Short Form Health Survey (SF-36), n (%)** |  |  |  |  |
| General health | 2.6 (0.8) | 2.5 (0.8) | 2.6 (0.8) | 2.4 (0.8) |
| Compared to one-year, general heath now | 3.3 (0.5) | 2.8 (1) | 2.7 (1.3) | 2.8 (1.2) |
| Pain interfered with normal work in the past 4 weeks | 2.8 (1) | 2.5 (0.9) | 2.9 (0.9) | 2.1 (0.6) |
| Physical health/emotional problems interfered with social activities in the past 4 weeks | 3.3 (1.4) | 3.4 (1.6) | 3.8 (1.3) | 3.5 (1.6) |
| **Health status: EuroQol EQ-5D 5L, n (%)** |  |  |  |  |
| Mobility |  |  |  |  |
| No problems | 1 (9.1%) | 4 (36.4%) | 5 (50%) | 4 (40%) |
| Slight | 4 (36.4%) | 4 (36.4%) | 2 (20%) | 5 (50%) |
| Moderate | 6 (54.5%) | 2 (18.2%) | 3 (30%) | 1 (10%) |
| Severe | 0 | 1 (9.1%) | 0 | 0 |
| Extreme | 0 | 0 | 0 | 0 |
| Self-care |  |  |  |  |
| No problems | 11 (100%) | 11 (100%) | 8 (80%) | 9 (90%) |
| Slight | 0 | 0 | 2 (20%) | 1 (10%) |
| Moderate | 0 | 0 | 0 | 0 |
| Severe | 0 | 0 | 0 | 0 |
| Extreme | 0 | 0 | 0 | 0 |
| Usual activities |  |  |  |  |
| No problems | 3 (27.3%) | 8 (72.7%) | 4 (40%) | 4 (40%) |
| Slight | 7 (63.6%) | 3 (27.3%) | 4 (40%) | 5 (50%) |
| Moderate | 1 (9.1%) | 0 | 2 (20%) | 1 (10%) |
| Severe | 0 | 0 | 0 | 0 |
| Extreme | 0 | 0 | 0 | 0 |
| Pain/Discomfort |  |  |  |  |
| No problems | 0 | 0 | 2 (20%) | 4 (40%) |
| Slight | 3 (27.3%) | 8 (72.7%) | 8 (80%) | 6 (60%) |
| Moderate | 6 (54.5%) | 2 (18.2%) | 0 | 0 |
| Severe | 2 (18.2%) | 1 (9.1%) | 0 | 0 |
| Extreme | 0 | 0 | 0 | 0 |
| Anxiety/Depression |  |  |  |  |
| No problems | 5 (45.5%) | 10 (90.1%) | 4 (40%) | 8 (80%) |
| Slight | 5 (45.5%) | 1 (9.1%) | 5 (50%) | 1 (10%) |
| Moderate | 1 (9.1%) | 0 | 1 (10%) | 1 (10%) |
| Severe | 0 | 0 | 0 | 0 |
| Extreme | 0 | 0 | 0 | 0 |
| VAS health today, M (SD) | 73.4 (9.4) | 75.5 (16.3) | 76.1 (15.1) | 78 (17.5) |
| **Sleep quality-PSQI, M (SD)** | 9.4 (2.7) | 8.9 (2.6) | 8.7 (2.8) | 9 (2.5) |
| **Fear and beliefs-KOFBeQ, total score, M (SD)** | 50.9 (18.4) | 57.8 (18.3) | 61.8 (27.2) | 62.6 (22.5) |
| Daily living | 17.7 (6.5) | 19.6 (6.4) | 18 (7.7) | 18.9 (5.8) |
| Physicians | 17.6 (10.9) | 19.7 (11.3) | 23.2 (10.5) | 22.6 (9.1) |
| Disease | 7.7 (5.9) | 9.5 (4.5) | 10.1 (5.1) | 10.7 (6.1) |
| Sports | 7.8 (5.2) | 9 (3.9) | 10.5 (6.6) | 10.4 (4.3) |
| **Attention and awareness to pain-PVAQ, M (SD)** | 38.9 (11.5) | 36.2 (14.8) | 33.5 (12.1) | 34.7 (7.3) |
| **Pain catastrophizing-PCS, total score, M (SD)** | 10.7 (10.5) | 11.2 (12.7) | 13.7 (8.4) | 12 (5.8) |
| PCS—rumination score | 3.5 (3.4) | 3.5 (4.5) | 4.4 (2.9) | 4.6 (2.3) |
| PCS—helplessness score | 4.2 (4.9) | 4.7 (5.3) | 5.4 (4.9) | 4.5 (3.4) |
| PCS—magnification score | 3.1 (2.6) | 2.9 (3.3) | 3.9 (2.1) | 2.9 (1.6) |
| **Self-efficacy- PSEQ-2, total score, M (SD)** | 10.7 (1.5) | 11.1 (1) | 10.6 (1.4) | 10.8 (1.3) |
| Need of help with confidence, n (%) | 0 | 0 | 0 | 0 |
| Self-efficacy/confident, n (%) | 11 (100%) | 11 (100%) | 10 (100%) | 10 (100%) |
| **Resilience-BRS, total score, M (SD)** | 3.4 (0.5) | 3.5 (0.7) | 3.4 (0.9) | 3.6 (0.7) |
| Low resilience, n (%) | 2 (18.2%) | 3 (27.3%) | 3 (30%) | 3 (30%) |
| Normal resilience, n (%) | 9 (81.8%) | 8 (72.7%) | 6 (60%) | 6 (60%) |
| High resilience, n (%) | 0 | 0 | 1 (10%) | 1 (10%) |
| **Coping Strategies-CSQ, M (SD)** |  |  |  |  |
| Adaptive strategies |  |  |  |  |
| Diverting Attention | 5.7 (3) | 5.7 (3.2) | 3 (3.4) | 3.3 (3.4) |
| Reinterpreting pain Sensations | 2.7 (2.6) | 3.2 (2.4) | 3.3 (3.6) | 4.8 (3) |
| Ignoring Sensations | 6.2 (1.8) | 6.6 (2.5) | 6.1 (3.2) | 7.2 (1.7) |
| Coping Self-Statements | 7.6 (1.9) | 8.3 (2.7) | 7.2 (3.6) | 8.1 (3.3) |
| Increased Behavioral Activities | 6.9 (3.3) | 7.4 (3.6) | 5.9 (2.2) | 6.2 (2.6) |
| Maladaptive strategies |  |  |  |  |
| Catastrophizing | 2.8 (2.4) | 2.8 (3.7) | 3.6 (3.1) | 2.7 (2.5) |
| Praying and hoping | 3 (3.4) | 3.5 (3.3) | 2.1 (2) | 1.8 (1.4) |
| **Pain acceptance- CPAQ, total score, M (SD)** | 30.1 (6.2) | 28.8 (3.8) | 29.5 (5.1) | 26.3 (10.1) |
| Activity engagement | 15.9 (3.1) | 16.4 (2.1) | 15.3 (2.7) | 14.6 (5.6) |
| Pain willingness | 14.2 (4.1) | 12.5 (3.4) | 14.2 (3.7) | 11.7 (4.8) |
| **Depression, Anxiety, and Stress -DASS, total score, M (SD)** | 11.1 (10.7) | 6.7 (4.8) | 10.6 (7.4) | 6.1 (4.1) |
| Depression score | 3.9 (3.6) | 2.6 (2.1) | 3.2 (2.9) | 1.6 (1.6) |
| Anxiety score | 2.8 (3.4) | 1 (1.2) | 3.2 (2.8) | 1.5 (1.6) |
| Stress score | 4.4 (4.5) | 3.1 (2.3) | 4.2 (2.5) | 3 (2.3) |
| **Central Sensitisation Inventory, total score, M (SD)** | 26.5 (10.4) | 21.3 (8.6) | 28.8 (10.1) | 23.5 (12.4) |
| CSI ≥ 40, n (%) | 1 (9.1%) | 1 (9.1%) | 2 (20%) | 2 (20%) |
| CSI ≤ 40, n (%) | 10 (90.1%) | 10 (90.1%) | 8 (80%) | 8 (80%) |

Alahmari, K., Silvian, S.P., Ahmad, I., Reddy, R.S., and Kakaraparthi, V.N. (2020). Subjective and objective evaluation of pain for older adults with knee osteoarthritis in Saudi Arabia: A reliability study. *Niger J Clin Pract* 23(7)**,** 934-943. doi: 10.4103/njcp.njcp_270_19.

Alschuler, K.N., Molton, I.R., Jensen, M.P., and Riddle, D.L. (2013). Prognostic value of coping strategies in a community-based sample of persons with chronic symptomatic knee osteoarthritis. *Pain* 154(12)**,** 2775-2781. doi: 10.1016/j.pain.2013.08.012.

Baranoff, J., Hanrahan, S.J., Kapur, D., and Connor, J.P. (2014). Validation of the Chronic Pain Acceptance Questionnaire-8 in an Australian pain clinic sample. *Int J Behav Med* 21(1)**,** 177-185. doi: 10.1007/s12529-012-9278-6.

Bartley, E.J., Hossain, N.I., Gravlee, C.C., Sibille, K.T., Terry, E.L., Vaughn, I.A., et al. (2019). Race/Ethnicity Moderates the Association Between Psychosocial Resilience and Movement-Evoked Pain in Knee Osteoarthritis. *ACR Open Rheumatol* 1(1)**,** 16-25. doi: 10.1002/acr2.1002.

Benhamou, M., Baron, G., Dalichampt, M., Boutron, I., Alami, S., Rannou, F., et al. (2013). Development and validation of a questionnaire assessing fears and beliefs of patients with knee osteoarthritis: the Knee Osteoarthritis Fears and Beliefs Questionnaire (KOFBeQ). *PLoS One* 8(1)**,** e53886. doi: 10.1371/journal.pone.0053886.

Bilbao, A., Garcia-Perez, L., Arenaza, J.C., Garcia, I., Ariza-Cardiel, G., Trujillo-Martin, E., et al. (2018). Psychometric properties of the EQ-5D-5L in patients with hip or knee osteoarthritis: reliability, validity and responsiveness. *Qual Life Res* 27(11)**,** 2897-2908. doi: 10.1007/s11136-018-1929-x.

Dobson, F., Hinman, R.S., Roos, E.M., Abbott, J.H., Stratford, P., Davis, A.M., et al. (2013). OARSI recommended performance-based tests to assess physical function in people diagnosed with hip or knee osteoarthritis. *Osteoarthritis Cartilage* 21(8)**,** 1042-1052. doi: 10.1016/j.joca.2013.05.002.

Dunn, K.M., and Croft, P.R. (2005). Classification of low back pain in primary care: using “bothersomeness” to identify the most severe cases. *Spine* 30(16)**,** 1887-1892.

Fish, R.A., McGuire, B., Hogan, M., Morrison, T.G., and Stewart, I. (2010). Validation of the Chronic Pain Acceptance Questionnaire (CPAQ) in an Internet sample and development and preliminary validation of the CPAQ-8. *PAIN* 149(3).

Gay, C., Guiguet-Auclair, C., Mourgues, C., Gerbaud, L., and Coudeyre, E. (2019). Physical activity level and association with behavioral factors in knee osteoarthritis. *Annals of Physical and Rehabilitation Medicine* 62(1)**,** 14-20. doi: <https://doi.org/10.1016/j.rehab.2018.09.005>.

Gervais-Hupé, J., Pollice, J., Sadi, J., and Carlesso, L.C. (2018). Validity of the central sensitization inventory with measures of sensitization in people with knee osteoarthritis. *Clinical Rheumatology* 37(11)**,** 3125-3132. doi: 10.1007/s10067-018-4279-8.

Goodin, B.R., Bulls, H.W., Herbert, M.S., Schmidt, J., King, C.D., Glover, T.L., et al. (2014). Temporal summation of pain as a prospective predictor of clinical pain severity in adults aged 45 years and above with knee osteoarthritis: ethnic differences. *Psychosomatic medicine* 76(4)**,** 302.

Gray, J., Kim, J., Ciesla, J.R., and Yao, P. (2016). Rasch Analysis of the Lubben Social Network Scale-6 (LSNS-6). *J Appl Gerontol* 35(5)**,** 508-528. doi: 10.1177/0733464814560468.

Harkins, S.W., Price, D.D., and Braith, J. (1989). Effects of extraversion and neuroticism on experimental pain, clinical pain, and illness behavior. *Pain* 36(2)**,** 209-218. doi: 10.1016/0304-3959(89)90025-0.

Hawker, G.A., Davis, A.M., French, M.R., Cibere, J., Jordan, J.M., March, L., et al. (2008). Development and preliminary psychometric testing of a new OA pain measure--an OARSI/OMERACT initiative. *Osteoarthritis Cartilage* 16(4)**,** 409-414. doi: 10.1016/j.joca.2007.12.015.

He, S., and Wang, J. (2017). Validation of the Social support and Pain Questionnaire (SPQ) in patients with painful temporomandibular disorders. *J Headache Pain* 18(1)**,** 57. doi: 10.1186/s10194-017-0766-6.

Herbert, M.S., Goodin, B.R., Pero, S.T.t., Schmidt, J.K., Sotolongo, A., Bulls, H.W., et al. (2014). Pain hypervigilance is associated with greater clinical pain severity and enhanced experimental pain sensitivity among adults with symptomatic knee osteoarthritis. *Ann Behav Med* 48(1)**,** 50-60. doi: 10.1007/s12160-013-9563-x.

Hochman, J.R., Davis, A.M., Elkayam, J., Gagliese, L., and Hawker, G.A. (2013). Neuropathic pain symptoms on the modified painDETECT correlate with signs of central sensitization in knee osteoarthritis. *Osteoarthritis Cartilage* 21(9)**,** 1236-1242. doi: 10.1016/j.joca.2013.06.023.

Hochman, J.R., Gagliese, L., Davis, A.M., and Hawker, G.A. (2011). Neuropathic pain symptoms in a community knee OA cohort. *Osteoarthritis Cartilage* 19(6)**,** 647-654. doi: 10.1016/j.joca.2011.03.007.

Imai, Y., Petersen, K.K., Mørch, C.D., and Arendt Nielsen, L. (2016). Comparing test–retest reliability and magnitude of conditioned pain modulation using different combinations of test and conditioning stimuli. *Somatosensory & motor research* 33(3-4)**,** 169-177.

Jakorinne, P., Haanpää, M., and Arokoski, J. (2018a). Reliability of pressure pain, vibration detection, and tactile detection threshold measurements in lower extremities in subjects with knee osteoarthritis and healthy controls. *Scand J Rheumatol* 47(6)**,** 491-500. doi: 10.1080/03009742.2018.1433233.

Jakorinne, P., Haanpää, M., and Arokoski, J. (2018b). Reliability of pressure pain, vibration detection, and tactile detection threshold measurements in lower extremities in subjects with knee osteoarthritis and healthy controls. *Scandinavian journal of rheumatology* 47(6)**,** 491-500.

Keller, S., Bann, C.M., Dodd, S.L., Schein, J., Mendoza, T.R., and Cleeland, C.S. (2004). Validity of the brief pain inventory for use in documenting the outcomes of patients with noncancer pain. *Clinical Journal of Pain* 20(5)**,** 309-318. doi: Doi 10.1097/00002508-200409000-00005.

Kennedy, D.L., Kemp, H.I., Ridout, D., Yarnitsky, D., and Rice, A.S.C. (2016). Reliability of conditioned pain modulation: a systematic review. *Pain* 157(11)**,** 2410-2419. doi: 10.1097/j.pain.0000000000000689.

Kleih, S., Riccio, A., Mattia, D., Kaiser, V., Friedrich, E., Scherer, R., et al. (2011). *Motivation influences performance in SMR-BCI.* na.

Kleih, S.C., and Kubler, A. (2013). Empathy, motivation, and P300 BCI performance. *Front Hum Neurosci* 7(642)**,** 642. doi: 10.3389/fnhum.2013.00642.

Kokkonen, M., and Pulkkinen, L. (2001). Examination of the paths between personality, current mood, its evaluation, and emotion regulation. *European Journal of Personality* 15(2)**,** 83-104. doi: DOI 10.1002/per.397.

Lewis, G.N., Heales, L., Rice, D.A., Rome, K., and McNair, P.J. (2012). Reliability of the conditioned pain modulation paradigm to assess endogenous inhibitory pain pathways. *Pain Res Manag* 17(2)**,** 98-102. doi: 10.1155/2012/610561.

Lluch Girbés, E., Dueñas, L., Barbero, M., Falla, D., Baert, I.A., Meeus, M., et al. (2016). Expanded distribution of pain as a sign of central sensitization in individuals with symptomatic knee osteoarthritis. *Physical therapy* 96(8)**,** 1196-1207.

Maddison, R., Ni Mhurchu, C., Jiang, Y., Vander Hoorn, S., Rodgers, A., Lawes, C.M., et al. (2007). International Physical Activity Questionnaire (IPAQ) and New Zealand Physical Activity Questionnaire (NZPAQ): a doubly labelled water validation. *Int J Behav Nutr Phys Act* 4(1)**,** 62. doi: 10.1186/1479-5868-4-62.

Mani, R., Adhia, D.B., Leong, S.L., Vanneste, S., and De Ridder, D. (2019). Sedentary behaviour facilitates conditioned pain modulation in middle-aged and older adults with persistent musculoskeletal pain: a cross-sectional investigation. *Pain Rep* 4(5)**,** e773. doi: 10.1097/PR9.0000000000000773.

Martina, I.S., van Koningsveld, R., Schmitz, P.I., van der Meche, F.G., and van Doorn, P.A. (1998). Measuring vibration threshold with a graduated tuning fork in normal aging and in patients with polyneuropathy. European Inflammatory Neuropathy Cause and Treatment (INCAT) group. *J Neurol Neurosurg Psychiatry* 65(5)**,** 743-747. doi: 10.1136/jnnp.65.5.743.

Mayer, J.D., and Gaschke, Y.N. (1988). The experience and meta-experience of mood. *J Pers Soc Psychol* 55(1)**,** 102-111. doi: 10.1037//0022-3514.55.1.102.

Mayer, T.G., Neblett, R., Cohen, H., Howard, K.J., Choi, Y.H., Williams, M.J., et al. (2012). The development and psychometric validation of the central sensitization inventory. *Pain Practice* 12(4)**,** 276-285.

McHorney, C.A., Ware, J.E., Jr., Lu, J.F., and Sherbourne, C.D. (1994). The MOS 36-item Short-Form Health Survey (SF-36): III. Tests of data quality, scaling assumptions, and reliability across diverse patient groups. *Med Care* 32(1)**,** 40-66. doi: 10.1097/00005650-199401000-00004.

Mendoza, T., Mayne, T., Rublee, D., and Cleeland, C. (2006). Reliability and validity of a modified Brief Pain Inventory short form in patients with osteoarthritis. *Eur J Pain* 10(4)**,** 353-361. doi: 10.1016/j.ejpain.2005.06.002.

Moore, R.L., Clifford, A.M., Moloney, N., Doody, C., Smart, K.M., and O’Leary, H. (2020). The Relationship Between Clinical and Quantitative Measures of Pain Sensitization in Knee Osteoarthritis. *The Clinical journal of pain* 36(5)**,** 336-343. doi: 10.1097/AJP.0000000000000798.

Musich, S., Wang, S.S., Slindee, L., Kraemer, S., and Yeh, C.S. (2019). Association of resilience and social networks with pain outcomes among older adults. *Population Health Management* 22(6)**,** 511-521.

Mutlu, E.K., and Ozdincler, A.R. (2015). Reliability and responsiveness of algometry for measuring pressure pain threshold in patients with knee osteoarthritis. *J Phys Ther Sci* 27(6)**,** 1961-1965. doi: 10.1589/jpts.27.1961.

Neblett, R., Hartzell, M.M., Cohen, H., Mayer, T.G., Williams, M., Choi, Y., et al. (2015). Ability of the central sensitization inventory to identify central sensitivity syndromes in an outpatient chronic pain sample. *Clin J Pain* 31(4)**,** 323-332. doi: 10.1097/AJP.0000000000000113.

Neogi, T., Frey-Law, L., Scholz, J., Niu, J., Arendt-Nielsen, L., Woolf, C., et al. (2015). Sensitivity and sensitisation in relation to pain severity in knee osteoarthritis: trait or state? *Ann Rheum Dis* 74(4)**,** 682-688. doi: 10.1136/annrheumdis-2013-204191.

Nicholas, M.K. (2007). The pain self-efficacy questionnaire: Taking pain into account. *Eur J Pain* 11(2)**,** 153-163. doi: 10.1016/j.ejpain.2005.12.008.

Nicholas, M.K., McGuire, B.E., and Asghari, A. (2015). A 2-item short form of the Pain Self-efficacy Questionnaire: development and psychometric evaluation of PSEQ-2. *J Pain* 16(2)**,** 153-163. doi: 10.1016/j.jpain.2014.11.002.

Nie, H., Arendt-Nielsen, L., Andersen, H., and Graven-Nielsen, T. (2005). Temporal Summation of Pain Evoked by Mechanical Stimulation in Deep and Superficial Tissue. *The Journal of Pain* 6(6)**,** 348-355. doi: 10.1016/j.jpain.2005.01.352.

Nijboer, F., Birbaumer, N., and Kübler, A. 2010. The influence of psychological state and motivation on brain-computer interface performance in patients with amyotrophic lateral sclerosis - a longitudinal study. *Frontiers in neuroscience* [Online], 4. Available: <http://europepmc.org/abstract/MED/20700521>

<https://www.ncbi.nlm.nih.gov/pmc/articles/pmid/20700521/pdf/?tool=EBI>

<https://www.ncbi.nlm.nih.gov/pmc/articles/pmid/20700521/?tool=EBI>

<https://doi.org/10.3389/fnins.2010.00055>

<https://europepmc.org/articles/PMC2916671>

<https://europepmc.org/articles/PMC2916671?pdf=render> [Accessed 2010].

Omachi, T.A. (2011). Measuring sleep in rheumatologic diseases: The ESS, FOSQ, ISI, and PSQI. *Arthritis care & research* 63(0 11)**,** S287.

Ong, W.J., Kwan, Y.H., Lim, Z.Y., Thumboo, J., Yeo, S.J., Yeo, W., et al. (2021). Measurement properties of Pain Catastrophizing Scale in patients with knee osteoarthritis. *Clin Rheumatol* 40(1)**,** 295-301. doi: 10.1007/s10067-020-05163-8.

Oppe, M., Devlin, N.J., and Szende, A. (2007). *EQ-5D value sets: inventory, comparative review and user guide.* Springer.

Panosyan, F.B., Mountain, J.M., Reilly, M.M., Shy, M.E., and Herrmann, D.N. (2016). Rydel-Seiffer fork revisited: Beyond a simple case of black and white. *Neurology* 87(7)**,** 738-740. doi: 10.1212/WNL.0000000000002991.

Peer, M.A., and Lane, J. (2013). The Knee Injury and Osteoarthritis Outcome Score (KOOS): a review of its psychometric properties in people undergoing total knee arthroplasty. *J Orthop Sports Phys Ther* 43(1)**,** 20-28. doi: 10.2519/jospt.2013.4057.

Price, D.D., Mcgrath, P.A., Rafii, A., and Buckingham, B. (1983). The Validation of Visual Analog Scales as Ratio Scale Measures for Chronic and Experimental Pain. *Pain* 17(1)**,** 45-56. doi: Doi 10.1016/0304-3959(83)90126-4.

Rheinberg, F., Vollmeyer, R., and Burns, B.D. (2001). FAM: Ein Fragebogen zur Erfassung aktueller Motivation in Lern-und Leistungssituationen (Langversion, 2001). *Diagnostica* 2**,** 57-66.

Riley, J.L., 3rd, Cruz-Almeida, Y., Staud, R., and Fillingim, R.B. (2019). Effects of manipulating the interstimulus interval on heat-evoked temporal summation of second pain across the age span. *Pain* 160(1)**,** 95-101. doi: 10.1097/j.pain.0000000000001382.

Roelofs, J., Peters, M.L., McCracken, L., and Vlaeyen, J.W.S. (2003). The pain vigilance and awareness questionnaire (PVAQ): further psychometric evaluation in fibromyalgia and other chronic pain syndromes. *Pain* 101(3)**,** 299-306. doi: 10.1016/S0304-3959(02)00338-X.

Ros, T., Enriquez-Geppert, S., Zotev, V., Young, K.D., Wood, G., Whitfield-Gabrieli, S., et al. (2020). "Consensus on the reporting and experimental design of clinical and cognitive-behavioural neurofeedback studies (CRED-nf checklist)". Oxford University Press).

Rosenberg, D.E., Norman, G.J., Wagner, N., Patrick, K., Calfas, K.J., and Sallis, J.F. (2010). Reliability and validity of the Sedentary Behavior Questionnaire (SBQ) for adults. *J Phys Act Health* 7(6)**,** 697-705. doi: 10.1123/jpah.7.6.697.

Severeijns, R., Vlaeyen, J.W., van den Hout, M.A., and Weber, W.E. (2001). Pain catastrophizing predicts pain intensity, disability, and psychological distress independent of the level of physical impairment. *Clin J Pain* 17(2)**,** 165-172. doi: 10.1097/00002508-200106000-00009.

Smith, B.W., Dalen, J., Wiggins, K., Tooley, E., Christopher, P., and Bernard, J. (2008). The brief resilience scale: assessing the ability to bounce back. *Int J Behav Med* 15(3)**,** 194-200. doi: 10.1080/10705500802222972.

Stanton, T.R., Lin, C.W., Smeets, R.J., Taylor, D., Law, R., and Lorimer Moseley, G. (2012). Spatially defined disruption of motor imagery performance in people with osteoarthritis. *Rheumatology (Oxford)* 51(8)**,** 1455-1464. doi: 10.1093/rheumatology/kes048.

Stanton, T.R., Lin, C.W.C., Bray, H., Smeets, R.J.E.M., Taylor, D., Law, R.Y.W., et al. (2013). Tactile acuity is disrupted in osteoarthritis but is unrelated to disruptions in motor imagery performance. *Rheumatology* 52(8)**,** 1509-1519. doi: 10.1093/rheumatology/ket139.

Starr, C.J., Sawaki, L., Wittenberg, G.F., Burdette, J.H., Oshiro, Y., Quevedo, A.S., et al. (2011). The contribution of the putamen to sensory aspects of pain: insights from structural connectivity and brain lesions. *Brain* 134(Pt 7)**,** 1987-2004. doi: 10.1093/brain/awr117.

Suwit, A., Rungtiwa, K., and Nipaporn, T. (2020). Reliability and Validity of the Osteoarthritis Research Society International Minimal Core Set of Recommended Performance-Based Tests of Physical Function in Knee Osteoarthritis in Community-Dwelling Adults. *Malays J Med Sci* 27(2)**,** 77-89. doi: 10.21315/mjms2020.27.2.9.

Thomas, M.J., Rathod-Mistry, T., Harper, S., Parry, E.L., Pope, C., Neogi, T., et al. (2019). Acute Flares of Knee Osteoarthritis (the ACT-FLARE Study): Protocol for a Web-Based Case-Crossover Study in Community-Dwelling Adults. *JMIR Res Protoc* 8(4)**,** e13428. doi: 10.2196/13428.

Tilley, P., and Bisset, L. (2017). The Reliability and Validity of Using Ice to Measure Cold Pain Threshold. *BioMed Research International* 2017**,** 7640649. doi: 10.1155/2017/7640649.

Uritani, D., Kasza, J., Campbell, P.K., Metcalf, B., and Egerton, T. (2020). The association between psychological characteristics and physical activity levels in people with knee osteoarthritis: a cross-sectional analysis. *BMC Musculoskelet Disord* 21(1)**,** 269. doi: 10.1186/s12891-020-03305-2.

Vader, K., Abebe, A.B., Chala, M.B., Varette, K., and Miller, J. (2020). Determining the feasibility of a trial to evaluate the effectiveness of phototherapy versus placebo at reducing pain during physical activity for people with knee osteoarthritis: a pilot randomized controlled trial. *Pilot and Feasibility Studies* 6(1)**,** 1-15. doi: ARTN 186

10.1186/s40814-020-00729-4.

Van Der Lugt, C.M., Rollman, A., Naeije, M., Lobbezoo, F., and Visscher, C.M. (2012). Social support in chronic pain: development and preliminary psychometric assessment of a new instrument. *J Oral Rehabil* 39(4)**,** 270-276. doi: 10.1111/j.1365-2842.2011.02269.x.

Webster, K.E., and Feller, J.A. (2016). Comparison of the short form-12 (SF-12) health status questionnaire with the SF-36 in patients with knee osteoarthritis who have replacement surgery. *Knee Surgery Sports Traumatology Arthroscopy* 24(8)**,** 2620-2626. doi: 10.1007/s00167-015-3904-1.

Wideman, T.H., Finan, P.H., Edwards, R.R., Quartana, P.J., Buenaver, L.F., Haythornthwaite, J.A., et al. (2014). Increased sensitivity to physical activity among individuals with knee osteoarthritis: Relation to pain outcomes, psychological factors, and responses to quantitative sensory testing. *PAIN* 155(4).

Wilkie, R., Jordan, J.L., Muller, S., Nicholls, E., Healey, E.L., and van der Windt, D.A. (2011). Measures of social function and participation in musculoskeletal populations: Impact on Participation and Autonomy (IPA), Keele Assessment of Participation (KAP), Participation Measure for Post-Acute Care (PM-PAC), Participation Objective, Participation Subjective (POPS), Rating of Perceived Participation (ROPP), and The Participation Scale. *Arthritis Care Res (Hoboken)* 63 Suppl 11**,** S325-336. doi: 10.1002/acr.20641.

Wilkie, R., Peat, G., Thomas, E., Hooper, H., and Croft, P.R. (2005). The Keele Assessment of Participation: a new instrument to measure participation restriction in population studies. Combined qualitative and quantitative examination of its psychometric properties. *Qual Life Res* 14(8)**,** 1889-1899. doi: 10.1007/s11136-005-4325-2.

Wood, B.M., Nicholas, M.K., Blyth, F., Asghari, A., and Gibson, S. (2010). The utility of the short version of the Depression Anxiety Stress Scales (DASS-21) in elderly patients with persistent pain: does age make a difference? *Pain Med* 11(12)**,** 1780-1790. doi: 10.1111/j.1526-4637.2010.01005.x.

Wylde, V., Palmer, S., Learmonth, I.D., and Dieppe, P. (2011). Test-retest reliability of Quantitative Sensory Testing in knee osteoarthritis and healthy participants. *Osteoarthritis Cartilage* 19(6)**,** 655-658. doi: 10.1016/j.joca.2011.02.009.

Yarnitsky, D., Bouhassira, D., Drewes, A.M., Fillingim, R.B., Granot, M., Hansson, P., et al. (2015). Recommendations on practice of conditioned pain modulation (CPM) testing. *Eur J Pain* 19(6)**,** 805-806. doi: 10.1002/ejp.605.

Youngcharoen, P., Aree-Ue, S., and Saraboon, Y. (2017). Validation of Pain Catastrophizing Scale Thai Version in Older Adults with Knee Osteoarthritis. *Innovation in Aging* 1(suppl_1)**,** 871-871. doi: 10.1093/geroni/igx004.3131.
